# Transposition favors the generation of large effect mutations that may facilitate rapid adaption

**DOI:** 10.1038/s41467-019-11385-5

**Published:** 2019-07-31

**Authors:** Leandro Quadrana, Mathilde Etcheverry, Arthur Gilly, Erwann Caillieux, Mohammed-Amin Madoui, Julie Guy, Amanda Bortolini Silveira, Stefan Engelen, Victoire Baillet, Patrick Wincker, Jean-Marc Aury, Vincent Colot

**Affiliations:** 1 Institut de Biologie de l’Ecole Normale Supérieure (IBENS), Centre National de la Recherche Scientifique (CNRS), Institut National de la Santé et de la Recherche Médicale (INSERM), Ecole Normale Supérieure, PSL Research University, Paris, 75005 France; 2 Genoscope, Institut de biologie François-Jacob, Commissariat à l’Energie Atomique (CEA), Universite Paris-Saclay, F-91057 Evry, France; 3 Genomique Metabolique, Genoscope, Institut de biologie François Jacob, CEA, CNRS, Universite d’Evry, Universite Paris-Saclay, 91057 Evry, France; 4 Present Address: Human Genetics, Wellcome Trust Sanger Institute, Hinxton, CB10 1SA UK; 5 Present Address: Translational Research Department, Institut Curie, PSL Research University, Paris, 75005 France

**Keywords:** DNA methylation, Plant evolution, Genetic variation, Mobile elements

## Abstract

Transposable elements (TEs) are mobile parasitic sequences that have been repeatedly coopted during evolution to generate new functions and rewire gene regulatory networks. Yet, the contribution of active TEs to the creation of heritable mutations remains unknown. Using TE accumulation lines in *Arabidopsis thaliana* we show that once initiated, transposition produces an exponential spread of TE copies, which rapidly leads to high mutation rates. Most insertions occur near or within genes and targets differ between TE families. Furthermore, we uncover an essential role of the histone variant H2A.Z in the preferential integration of *Ty1/copia* retrotransposons within environmentally responsive genes and away from essential genes. We also show that epigenetic silencing of new *Ty1/copia* copies can affect their impact on major fitness-related traits, including flowering time. Our findings demonstrate that TEs are potent episodic (epi)mutagens that, thanks to marked chromatin tropisms, limit the mutation load and increase the potential for rapid adaptation.

## Abstract (web summary)

The contribution of transposable elements (TEs) to the creation of heritable mutations is unknown. Here the authors show in Arabidopsis that TEs accumulate exponentially once mobilized and that *COPIA* retrotransposons preferentially integrate in environmental response genes in a H2A.Z-dependent manner.

## Introduction

Transposable elements (TEs) are ubiquitous parasitic DNA sequences that self-propagate  across genomes. Eukaryotic TEs fall into two broad classes: DNA transposons, that use a cut and paste mechanism for their mobilization, and retrotransposons, that move via a reverse transcribed RNA intermediate. TEs can be further subdivided into superfamilies and families based on specific sequence features^1^. Most TE sequences present in eukaryotic genomes are inactive remnants of once active copies^2,3^ and variation in the total content of these molecular fossils account for much of the large differences in genome size observed even among closely related species^4^. Moreover, TE sequences are recurrently involved during evolution in the rewiring of gene regulatory networks, as well as the creation of new cellular functions^5–7^.

Despite the importance of TEs as generators of evolutionary novelty, the range and phenotypic consequences of the heritable mutations produced through TE mobilization remain largely unknown^4,8^. One explanation is that TE insertion mutations are difficult to detect using short-read sequencing technologies, unlike the small-scale mutations typically generated by replication errors and DNA damage. Furthermore, because of the epigenetic mechanisms that keep TEs in check, including DNA methylation in plants and mammals^9^, spontaneous transposition is usually rare. Indeed, no TE-induced mutations were recovered in an in-depth analysis of *Arabidopsis thaliana* mutation accumulation (MA) lines^10,11^, in which small-scale mutations accumulate at a rate of approximately one per haploid genome per generation.  Also, as most TE insertions are likely subjected to strong purifying selection, population genomic analyses of polymorphic TE insertions provide a distorted view of TE mobilization^4,8,12^ and cannot easily assess potential integration biases.

To circumvent these limitations, we use a population of *Arabidopsis thaliana* epigenetic recombinant inbred lines (epiRILs). As a result of one of the two isogenic founder plants being deficient in the epigenetic silencing of TEs via DNA methylation^13^, transposition is ongoing for several TEs in the epiRILs, which are operationally similar to MA lines. Thus, the epiRILs allow a direct assessment of transposition-induced heritable mutations, unaltered by the filtering effect of natural selection. Our results reveal that thanks to specific chromatin tropisms, TE mobilization can rapidly generate high rates of mutations in fitness-related genes, while minimizing the mutation load.

## Results

### TE insertions accumulate in epiRILs

**Fig. 1:**
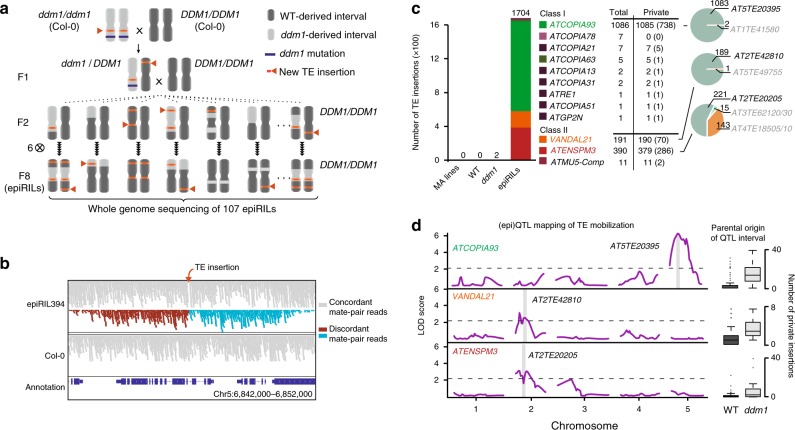
TE insertions accumulate in the epiRILs. **a** Crossing scheme used to generate the epiRIL population. **b** Sequencing alignment tracks for epiRIL 394 and Col-0. Concordant and discordant mate-pair reads, as well as the sense or antisense orientation for the latter, are indicated in gray, brown and cyan, respectively **c** Number and identity of insertions accumulated in wild-type, *ddm1* and the epiRILs. Number of hemizygous insertions are indicated in brackets. Pie charts show the proportion of insertions matching different full-length reference TE sequences. **d** (epi)QTL mapping of the number of insertions produced by *ATCOPIA93, VANDAL21*, and *ATENSPM3*. The full-length reference TE sequence located within the single (epi)QTL interval in each case is indicated. Box-plots indicate the variation in the number of insertions in the epiRILs in relation to the parental origin of the relevant (epi)QTL interval. For each boxplot, the lower and upper bounds of the box indicate the first and third quartiles, respectively, and the center line indicates the median. Source data of Figs. 1b and 1c are provided as a Source Data file

We previously produced a large population of *A. thaliana* epiRILs from an initial cross between two isogenic individuals, one of which carried a mutant allele of the epigenetic silencer gene *DDM1*, which is required for maintaining DNA methylation and silencing of TEs^14^. A single F1 individual was backcrossed to the wild-type parental line and homozygous F2 *DDM1/DDM1* progeny were propagated by repeated selfing and single seed descent through another six generations^13^ (Fig. 1a). Genome-wide analysis of DNA methylation revealed that approximately one third of the differentially methylated regions (DMRs) between the *ddm1* and wild-type parents are transmitted as such in the epiRILs^13,15^, which have each inherited on average 25% of their genome from the *ddm1* parent (Fig. 1a) and have therefore mosaic epigenomes. Thus, the epiRIL population constitutes an ideal system to study TE mobilization in an essentially wild-type context and to compare insertion patterns in chromosomal intervals derived from the *ddm1* and wild-type parents. Moreover, in this setting, heritable TE insertions should accumulate neutrally through random segregation, i.e., in the near absence of natural selection, which makes the epiRILs operationally similar to MA lines^10,16–18^, except for the initial TE reactivation induced by *ddm1*.

We obtained genome sequencing data for 107 epiRILs at generation F8 together with close relatives of the two founder plants, using Illumina mate-pair libraries, and the sequence reads were analyzed to identify non-reference (i.e., de novo) TE insertions. The large physical distance (~5 kb) between mate-pair reads enabled us to determine the complete sequence of the insertions, and thus of the donor TEs (Fig. 1b and Supplementary Fig. 1a). No de novo TE insertions were detected in the two wild-type siblings sequenced, nor in five *A. thaliana* MA lines that were derived from wild-type plants^10^, consistent with the low frequency of spontaneous TE mobilization^12^. In contrast, two non-reference TE insertions were detected in the sequenced *ddm1* individual and 95% of the 107 epiRILs harbored at least one de novo insertion (Supplementary Fig. 1b). The number of new insertions varied greatly between epiRILs, with a maximal value of 97 new insertions in one line. Almost all new insertions (98.7%) were private (i.e., present in a single epiRIL), indicating that they occurred during the propagation of the epiRILs rather than in the *ddm1* parent line (Fig. 1a). In addition, 1107 of the 1670 private insertions detected in total were found in the hemizygous state (Fig. 1c), indicating that transposition is likely still ongoing in the epiRILs at generation F8.

The LTR-retrotransposon family *ATCOPIA93* and the two DNA transposon families *ATENSPM3* and *VANDAL21*, which are among the most active in nature^12^, contributed over 95% of all private insertions (64.4%, 22.5%, and 11.2%, respectively), and another eight TE families contributed the rest (Fig. 1c). Moreover, 99% of the *ATCOPIA93* and *VANDAL21* insertions, as well as 58% of the *ATENSPM3* insertions were identical to or best-matched just one of the several cognate full-length sequences present in the reference genome (Fig. 1c). In the case of *ATCOPIA93*, this mobile copy is *Évadé* (*EVD*) consistent with previous findings^19,20^. Conversely, most of the remaining *ATENSPM3* insertions derived from two composite elements that do not encode a recognizable transposase (Supplementary Fig. 1c) and which therefore were presumably mobilized in *trans* by the single mobile full-length *ATENSPM3*.

To test whether most private insertions were generated from a single mobile reference sequence, we performed (epi)QTL mapping based on the hundreds of parental DMRs segregating in the epiRILs and which can therefore serve as bona-fide genetic markers^21,22^. For each of the three TE families, a single (epi)QTL interval was associated with the variation in the number of insertions between epiRILs (Fig. 1d) and this interval contained the single full-length reference TE sequence identified above. Incidentally, the lack of additional (epi)QTL intervals indicated that no other regions affected by *ddm1* contributed appreciably to TE mobilization, thus revealing a simple genetic architecture for TE mobilization in each case.

### Triggering of TE mobilization leads to exponential spread

**Fig. 2:**
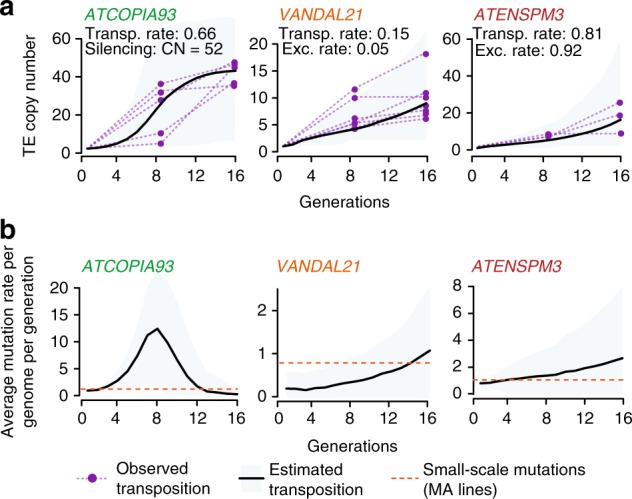
Transposition follows a chain reaction in the epiRILs. **a** Dynamics of insertion accumulation for the three TE families. Observed data obtained at F8 and F9 for ten epiRILs is shown for epiRILs harboring new private insertions. **b** Average rate across generations of TE-induced (black line) and small size mutations (red line) obtained using the epiRILs and MA lines^10^, respectively (bottom panels). Gray area represents 95% C.I. Transposition and excision rates per copy per generation, as well as TE copy number (CN) required for triggering concerted epigenetic silencing are indicated

To investigate the dynamics of TE mobilization, we carried out mathematical simulations based on a transposition-genetic drift scenario, assuming either that only the reference copy is mobile (master gene model) or that the reference and daughter copies are equally mobile (transposon model)^23^. In addition, our simulations incorporated three key parameters (Supplementary Fig. 2a), namely the rates of transposition and excision, as well as copy number-dependent inhibition of transposition. For all three TE families, the best fit to the observed data at generation F8 was obtained under a transposon model (Supplementary Fig. 2b). In this situation, insertion rates ranged between 0.15–0.81 per donor per generation, depending on the TE family (Fig. 2a and Supplementary Fig. 2c). These values are close to the rate of spontaneous point and small indel mutations per genome per generation in classical MA lines^10^. Thus, our results indicate that transposition is followed by an exponential spread of novel TE copies throughout the genome at rates that rapidly exceed those of small-size mutations (Fig. 2b).

To obtain direct evidence that new TE insertions as well as the initial donor TE could transpose in the epiRILs, we took advantage of the fact that TEs can acquire mutations during the transposition process^24^ to look for mutations that are shared by two or more private TE copies within a single epiRIL, as this pattern of sharing likely reflects consecutive mobilization events. While all *VANDAL21* private insertions were identical to the mobile reference element and were therefore not informative in this respect, approximately 5% of those produced by *ATENSMP3* and *ATCOPIA93* contained large internal deletions and small size mutations, respectively. Moreover, nine of these mutations were shared by two or more private insertions per epiRIL and sequencing of ten epiRILs advanced to generation F16 revealed in several cases an increase in the number of *ATENSPM3* private insertions sharing the same mutation (Supplementary Fig. 3a). These observations thus confirm that new TE copies are also mobile.

Although mobilization of DNA transposons involves excision, double-stranded gap repair can be used to replace the excised copy with that present on the homolog or the sister chromatid in case of post-replicative mobilization^25^. Such repair mechanism would thus lead to an apparent lack of excision, as well as a net increase in copy number and indeed our modeling predicted a low rate of excision (0.05 excision per transposition event) for *VANDAL21*. In agreement with this prediction, and consistent with observations in maize for the related *Mu* TE family^26^, the mobile *VANDAL21* reference copy was still present at its original position in most of the epiRILs. In contrast, our modeling predicted a high rate of excision (0.92 excision per transposition event) for *ATENSPM3* and we found that the mobile *ATENSPM3* reference copy was systematically lost in the epiRILs that inherited the relevant genomic region from the *ddm1* parent (Supplementary Fig. 2d). Moreover, numerous small indels compatible with *ATENSPM3* excision footprints were detected in the epiRILs, revealing that once mobilized, *ATENSMP3* rapidly becomes a major source of small indels in the *A. thaliana* genome (Supplementary Fig. 2e, f).

Our mathematical simulations indicated also that by F12, transposition should stop for *ATCOPIA93* in most epiRILs. Specifically, the number of new *ATCOPIA93* copies was expected to plateau at around 50 copies per diploid genome after 6–14 generations (Fig. 2a), which would be consistent with previous experimental data showing that for this TE, siRNA-mediated epigenetic silencing is initiated beyond a threshold of about 40 new copies^20^. To test this aspect of the modeling, we analyzed the genome sequence obtained for the ten epiRILs advanced to generations F16. *ATCOPIA93* insertions continued to accumulate between F8 and F16 in the five epiRILs that showed *ATCOPIA93* mobilization at F8 but not in the others. All five epiRILs with mobile *ATCOPIA93* harbored around 50 copies at F16 and in four of these epiRILs, all copies were methylated and silenced (Supplementary Fig. 3b). Furthermore, finer grain analysis for one epiRIL (epiRIL394) indicates that all *ATCOPIA93* copies underwent within one generation an ‘all-or-nothing’ gain of DNA methylation, thus resulting in their concomitant silencing (Supplementary Fig. 3c), as was reported before for two other epiRILs^20^. Taken together, these findings reveal that once active, TEs can rapidly become the major source of heritable mutations in the genome, until epigenetic silencing sets in to prevent further transposition, which in the case of *ATCOPIA93* happens at once in a copy-dependent manner after a few generations.

### Active TEs preferentially target genes

Overall, private insertions for *VANDAL21, ATENSPM3*, and *ATCOPIA93* were distributed evenly across the chromosomes (Supplementary Fig. 4a). Nonetheless, because the epiRILs have mosaic epigenomes^22^, potential differences in insertion frequency could exist between chromosomal intervals of different parental origin. Indeed, for all three TE families, the percentage of insertions in pericentromeric regions was significantly higher when these were derived from the *ddm1*  parent (Supplementary Fig. 4a). As pericentromeres lose some of their heterochromatic features in an heritable manner in *ddm1*^14,22,27^, we conclude that euchromatin is the preferred substrate for the integration of *VANDAL21, ATENSPM3*, and *ATCOPIA93*.

**Fig. 3:**
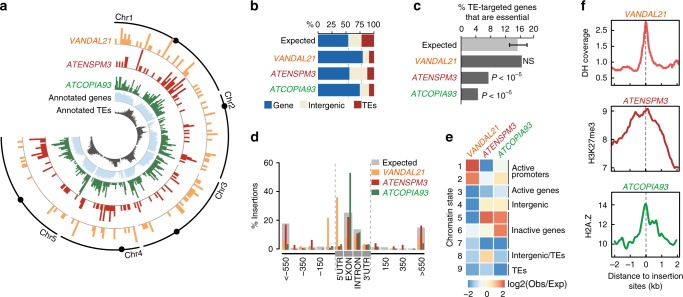
TEs exhibit strong and diverse chromatin-associated insertion biases towards genes. **a** Circos representation of private TE insertions detected for *VANDAL21*, *ATENSPM3*, and *ATCOPIA93* within wild-type intervals. Centromere positions are indicated by black dots. **b** Fraction of TE insertions in genes, TEs and intergenic regions. **c** Fraction of essential genes among genes targeted by *VANDAL21*, *ATENSPM3* or *ATCOPIA93* in the epiRILs. Statistical significance for each comparison was obtained using the Chi-square test. **d** Metagene analysis showing the distribution of new insertions. **e** Relative abundance of insertion sites in relation to the nine chromatin states defined in *A. thaliana*. **f** Levels of DNAse hypersensitivity (DH; top panel), H3K7me3 (middle panel), and H2A.Z (bottom panel) around *VANDAL21*, *ATENSPM3*, and *ATCOPIA93* insertion sites, respectively. Source data are provided as a Source Data file

With few exceptions, DNA methylation and transcription do not differ detectably between the wild-type derived intervals of the epiRILs and the corresponding intervals of wild-type plants^22,28^. We therefore only considered the wild-type derived intervals of the epiRILs  in our subsequent analyses, as they enabled us to obtain a genome-wide view of TE integration patterns that is unaffected by the epigenetic changes caused by *ddm1*. Most insertions were located preferentially along the chromosome arms (Fig. 3a) and they were strongly enriched near or within genes in the case of *VANDAL21* and *ATCOPIA9*3 (Fig. 3b). In addition, while known or predicted essential genes^29^ were targeted as frequently as other protein coding genes by *VANDAL21*, they were underrepresented among *ATENSPM3* and *ATCOPIA93* gene targets (Fig. 3c), suggesting that these last two TEs either avoid non-essential genes or tend to produce strong deleterious effects, or both. Consistent with the first possibility, hemizygous insertions within essential genes were also underrepresented, a result not expected if these insertions created recessive, loss-of-function alleles. For *ATCOPIA93*, strong deleterious effects were also manifest, as almost none of the insertions within essential genes were in the homozygous state. The situation was different for *ATENSPM3* insertions within essential genes, which were more often in the homozygous than the hemizygous state (Supplementary Fig. 4b). This last observation suggested that *ATENSPM3*, which excised frequently in the epiRILs, produced stronger deleterious effects upon excision than upon integration. Indeed, none of 98 homozygous *ATENSPM3* excision footprints in the epiRILs were located within essential genes (Supplementary Fig. 4b). Why *ATENSPM3* should be more deleterious upon excision remains to be determined.

### TEs targets have specific chromatin signatures

Although *VANDAL21*, *ATENSPM3*, and *ATCOPIA93* inserted preferentially near or within genes, their integration patterns differed markedly in relation to several features. Thus, *VANDAL21* mainly targeted the promoters and 5’ UTRs of broadly active genes, which are associated with two of the nine chromatin states defined in *A. thaliana*^30^ (states 1 and 2, Fig. 3d, e). These two states are enriched in the active marks H3K4me3 and H3K36me3. In contrast, *ATENSPM3* and *ATCOPIA93* preferentially targeted genes that tend to be repressed and enriched throughout their body in the histone mark H3K27me3, as well as the histone variant H2A.Z (chromatin state 5), which confers reduced stability to nucleosomes^31^. Furthermore, *ATCOPIA93* insertions were also overrepresented in genes whose body is solely enriched in H2A.Z (chromatin state 6, Fig. 3e). Meta-analyses confirmed these findings and indicated that *VANDAL21* insertion sites coincided with local peaks of DNase I hypersensitivity (Fig. 3f) that correspond to the transcriptional start site (TSS) of genes (Supplementary Fig. 4c), in keeping with previous observations^32^. In contrast, *ATENSPM3* and *ATCOPIA93* insertion sites were more broadly distributed over genes (Fig. 3d) with a marked preference for exons in the case of *ATCOPIA93.* Insertion sites for this TE were also characterized by local maxima of nucleosomal occupancy and H2A.Z enrichment (Supplementary Fig. 4d-f). We conclude that the integration patterns for *VANDAL21*, *ATENSPM3,* and *ATCOPIA93* are highly skewed towards distinct sets of genes and associated with specific chromatin states.

### H2A.Z guides integration of *Ty1/copia*

To gain further mechanistic insights into TE insertion preferences, we focused on the association between H2A.Z and *ATCOPIA93* insertion sites. In *A. thaliana*, when DNA methylation is compromised, hypomethylated TE sequences tend to acquire arrays of H2A.Z-containing nucleosomes^33^ and in the epiRILs, annotated TE sequences were more often hit by *ATCOPIA93* when inherited from the *ddm1* parent  (Supplementary Fig. 4g). The relocalization of *ATCOPIA93* insertions towards hypomethylated TE sequences provided a first indication that chromatin enriched in H2A.Z may play a role in the integration preference of *ATCOPIA93*.

**Fig. 4:**
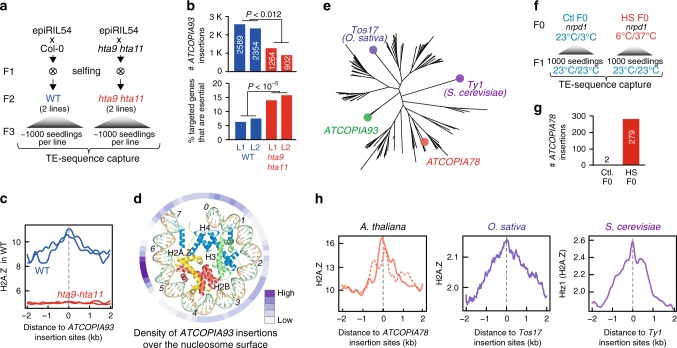
H2A.Z guides the integration of *Ty1/Copia* retrotransposons. **a** Experimental strategy for determining the role of H2A.Z in the integration of *ATCOPIA93*. **b** Number of new *ATCOPIA93* insertions detected in *HTA9 HTA11* and *hta9 hta11* F3 seedlings (top). Fraction of essential genes among those targeted by *ATCOPIA93* (bottom). Statistically significant differences were calculated using the chi-square test. **c** Meta-analysis of levels of H2A.Z levels around *ATCOPIA93* insertion sites. **d** Density of *ATCOPIA93* insertions over well positioned nucleosomes. **e** Phylogenetic analysis of *Tos17*, *Ty1 and* 110*A. thaliana Ty1/Copia* LTR-retrotransposons. **f** Experimental strategy for studying transposition of the heat-responsive *ATCOPIA78* LTR-retrotransposon in *A. thaliana*. **g** Number of new *ATCOPIA78* insertions detected in F1 seedlings of *nrpd1* plants grown under control or heat-stress conditions. **h** Meta-analysis of *A. thaliana*, rice (*O. sativa*) and yeast (*S. cerevisiae*) H2A.Z levels around *ATCOPIA78*, *Tos17*, and *Ty1* insertion sites, respectively. For *A. thaliana*, experimental and natural insertions are indicated by solid and dotted lines, respectively. Source data of Figure panels. **b**, **d**, **g**, and **h** are provided as a Source Data file

To test causality directly, we used one epiRIL to introduce through crosses several active *ATCOPIA93* copies into mutant plants that lack most H2A.Z^34^ (*hta9 hta11* genotype) as well as into wild-type plants (Fig. 4a). Heritable insertions produced by the introgressed *ATCOPIA93* copies were identified by TE-sequence capture^12^ in 1000 seedlings derived from two individuals of each genotype. Consistent with the transposition rate estimated in the epiRILs and with heritable insertions being produced late during flower development^20^, a total of 2354 and 2589 insertions were identified in the two pools of wild-type seedlings. In contrast, we recovered only 902 and 1264 insertions in pools of mutant seedlings (Fig. 4b), indicating that H2A.Z is required for *ATCOPIA93* mobilization. Furthermore, the strong *ATCOPIA93* integration preferences observed in the wild-type intervals of the epiRILs, as well as in the wild-type seedlings, were fully abolished in the mutant seedlings (Fig. 4c and Supplementary Fig. 5a). As a result of the more uniform transposition landscape obtained in mutant seedlings, there were almost three times as many essential genes targeted by *ATCOPIA93* in these plants compared to the wild-type (Fig. 4b). Together, these results reveal that by targeting H2A.Z-containing nucleosome arrays, *ATCOPIA93* avoids essential genes and thus minimizes the risks of being lost together with its host.

The large number of *ATCOPIA93* insertions detected in wild-type seedlings enabled us to examine potential integration preferences at the sub-nucleosomal level. Using well-positioned nucleosomes^35^, a major peak of integration was observed ~55 bp away from the nucleosome dyad (Fig. 4d). This position is one of the main points of contact between DNA and H2A or H2A.Z and it is also where these two histones diverge by several key amino acids^36^, thus providing further support for a direct role of H2A.Z in guiding *ATCOPIA93* integration.

To determine if H2A.Z plays a similar guiding role for other TEs of the *Ty1/copia* superfamily, we examined *ATCOPIA78* (also known as *ONSEN*^37^), which is distantly related to *ATCOPIA93* (Fig. 4e). There were no private *ATCOPIA78* insertions in the epiRILs, but previous work showed that *ATCOPIA78* can be transcriptionally reactivated by heat stress and also mobilized in stressed plants defective in so-called RNA-directed DNA methylation, such as in the mutant *nrpd1*^37^. We therefore assessed the mobilization of *ATCOPIA78* by TE sequence capture, using genomic DNA extracted from the progeny of pools of *nrpd1* plants that were subjected to heat stress at the seedling stage (Fig. 4f). A total of 279 new insertions were recovered in this way (Fig. 4g), which revealed targeting preferences similar to those of *ATCOPIA93* (Fig. 4h and Supplementary Fig. 5b-d). Furthermore, we previously identified 147 recent (i.e., low frequency, non-reference) *ATCOPIA78* insertions in the genome sequence of 211 natural *A. thaliana* accessions^12^ and the distribution of these natural insertions resembled that determined experimentally for *ATCOPIA78*, including the preference for H2A.Z containing chromatin (Fig. 4h). We also  examined the integration patterns of *Ty1/Copia* retrotransposons in relation to H2A.Z in the distantly related plant species *O. sativa* (rice)^38^, as well as in the yeast *S. cerevisiae*^39^ and found that in these two species, experimentally induced insertions were also located at sites enriched in H2A.Z (Fig. 4h). Taken together, these results suggest that the role of H2A.Z in the integration of *Ty1/Copia* retrotransposons has been evolutionarily conserved since the last common ancestor of plants and fungi.

### *ATCOPIA* preferentially target environmentally responsive genes

**Fig. 5:**
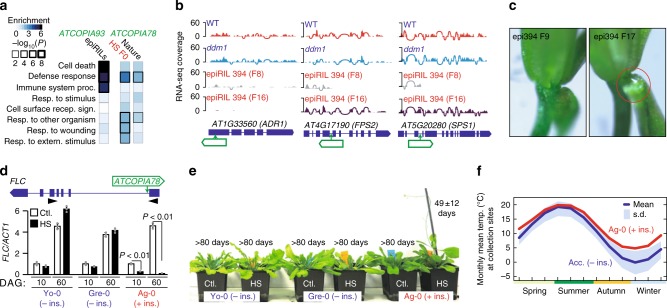
*ATCOPIA* preferentially targets environmentally responsive genes. **a** GO term analysis of genes with *ATCOPIA93* or *ATCOPIA78* insertions in the epiRILs or other experimental settings, or in nature. **b** Genome browser view of RNA-seq coverage of three genes hit by *ATCOPIA93* in epiRIL394. **c**. Nectar secretion in flowers of epiRIL394 at F9 and F17. A nectar droplet is only observed at F17 (circle). **d** Structure of the *FLC* locus with the position of the *ATCOPIA78* insertion in accession Ag-0 (top). Relative expression level of *FLC* at 10 and 60 days after germination (DAG) in plants containing (red) or lacking (blue) the *ATCOPIA78* insertion and grown under control conditions (ctl.) or subjected to heat stress (HS) at the seedling stage (bottom). Data are mean ± s.d. (*n* > 9 independent samples, one biological experiment) and statistical significance for differences was obtained using the MWU test. **e**. Flowering time (mean ± s.d.; *n* > 9 independent samples, two independent experiments) for the same plants as in **d**. **f** Monthly mean temperature at collection sites for Ag-0 and 25 accessions sharing the same *FLC* haplotype but lacking the *ATCOPIA78* insertion. Source data of Figure panels **d** and **e** are provided as a Source Data file

The deposition of H2A.Z within gene bodies is specific to plants and predominantly concerns environmentally responsive genes^40^, which were substantially overrepresented among *ATCOPIA93* and *ATCOPIA78* targets (Fig. 5a). To investigate the phenotypic impact of such targeting, we performed RNA-seq on pools of seedlings at generation F8 and F16 for one epiRIL (epiRIL 394) that contained five homozygous *ATCOPIA93* insertions at generation F8 within genes, including four that are environmentally responsive. Specifically, one insertion was within the second exon of the gene *AT5G38940* involved in salt stress and a second insertion was within the 5’UTR of the gene *ADR1* (*AT1G33560)*, which encodes an NBS-LRR disease resistance protein. The remaining two insertions were intronic and affected genes *FPS2* (*AT4G17190*) and *SPS1* (*AT5G20280*), which are implicated in defense against aphids^41^ and nectar secretion^42^, respectively. RNA-seq data were compared with those obtained for wild-type as well as *ddm1* seedlings and we found that all four genic insertions were associated with reduced transcript levels of their target at F8 (Fig. 5b). However, results differed for generation F16, when epiRIL 394 contained a total of  40 methylated copies of *ATCOPIA93* per diploid genome (i.e., 19 distinct new insertions in the homozygous state in addition to *EVD*; Supplementary Fig. 3b). While expression levels were reduced further for the 5’UTR insertion within *ADR1*, the transcript truncations associated with the two intronic insertions were fully abolished and both genes regained normal expression (Fig. 5b). These observations demonstrate that the effects of genic *ATCOPIA93* insertions can differ substantially depending on their precise location and epigenetic status. To gain further insight into potential phenotypic consequences, we examined nectar secretion, an adaptive trait that can be readily assessed in the laboratory and that could influence the degree of outcrossing in natural *A. thaliana* populations^43^. In agreement with the molecular data, nectar droplets were more conspicuous in flowers of epiRIL394 at generation F17 than at generation F9 (Fig. 5c).

We last investigated a natural *ATCOPIA78* insertion located within the first intron of *FLC* (Fig. 5d), which encodes a key repressor of flowering and is the main genetic determinant of flowering time variation between accessions^44^. This insertion is present in Ag-0 but absent from most other accessions examined^12^ (Supplementary Fig. 6), suggesting that it was generated in the recent past. We grew Ag-0 along with two other accessions (Yo-0 and Gre-0) with the same *FLC* haplotype, which contains a common polymorphism associated with vernalization response^45^, but lacking the *ATCOPIA78* insertion. All three accessions were late flowering (>80 days after germination, DAG) and had similar *FLC* expression under standard growth conditions. However, when subjected to heat stress at the seedling stage, Ag-0 flowered much earlier (49 DAG), unlike Yo-0 and Gre-0, which remained late flowering (Fig. 5e). Consistent with these observations, *FLC* expression remained unchanged in these two accessions following heat stress but was markedly reduced in Ag-0 (Fig. 5d), presumably as a result of the transient epigenetic reactivation of *ATCOPIA78*. Whether the stable silencing of *FLC* relies on the PRC2-based memory system implicated in the vernalization response^46^ or on other mechanisms remains to be determined. This notwithstanding, the fact that Ag-0 was collected from a site (Southwest of France) with much warmer winters than the other accessions that share the same *FLC* haplotype (Fig. 5f), suggests a role for the *ATCOPIA78* insertion in the local adaptation of Ag-0 to its environment, by enabling it to flower early even in the absence of vernalization.

## Discussion

Here, we have provided a direct assessment, unhindered by the filter of natural selection, of the spectrum, genomic distribution and phenotypic impact of heritable mutations generated by TE mobilization. We have shown that once initiated, transposition follows an exponential increase that leads to the accumulation of insertions at rates that rapidly exceed those of classical spontaneous mutations. Moreover, these much higher mutation rates are further accentuated as a result of pronounced, chromatin-associated integration preferences towards specific gene loci, which result in a dramatic narrowing down of the mutation target. Indeed, the finding that plant *Ty1/Copia* retrotransposons preferentially insert within environmentally responsive genes indicates that it is the primary cause of the high load of such insertions found in nature^12,47,48^.

We have also shown that the role of H2A.Z in the integration of *Ty1/Copia* retrotransposons is likely conserved from yeast to plants. This finding, combined with the previous observation that H2A.Z is enriched in the body of environmentally responsive genes in plants^33^, suggests that *Ty1/Copia* retrotransposons have been turned into epigenetically and environmentally sensitive engines of potential adaptive innovation specifically in  this group of organisms. In turn, this hypothetical scenario provides a plausible explanation for the invasive success of *Ty1/Copia*  retrotransposons specifically in the plant kingdom^4^.

The deep conservation across the tree of life of *Ty1/Copia* insertion preference for H2A.Z-containing nucleosomes contrasts with the observation that a close relative of *ATCOPIA93* preferentially inserts within centromeric repeats, which lack H2A.Z^49^, in its native host *A. lyrata*, as well as when introduced in *A. thaliana*^50^. Thus, integration specificity can nonetheless evolve remarkably fast at least for some *Copia* elements.

Given our findings were obtained using an experimental population of epiRILs, a key priority for the future will be to assess the frequency of *Ty1/Copia* retrotransposition episodes in nature and to identify the factors involved. In this regard, we note that *ATCOPIA78* as well as other *ATCOPIA* families have accumulated more copies in *A. thaliana* accessions that have colonized central Asia, which is characterized by extreme temperatures shifts between seasons^12^. Thus, *ATCOPIA* mobilization could be more prevalent in times of crisis, such as during the colonization of novel habitats and limited stading genetic variation. Were this the case, populations experiencing such retrotransposition episodes would increase their genetic diversity in a manner that could facilitate their rapid adaptation to the new conditions encountered. By extension, the modulation of integration preferences through chromatin perturbations could provide a viable strategy to harness *Ty1/Copia* mobilization for the creation of adaptive alleles in the face of a changing climate, notably in crops with limited genetic diversity.

In summary, by demonstrating that TE mobilization generates large effect mutations non-uniformly across the genome and generations, our work challenges the assumption that the evolutionary process is solely fueled by random mutations arising at a slow rate in a clockwise manner.

## Methods

### Samples and experimental model

The following *A. thaliana* plants were used: wild-type Col-0, Ag-0, Yo-0 and Gre-0, obtained from Versailles collection. The Col-0 *ddm1-2* mutant and the epiRILs population^13^, the Col-0 *nrpd1-3* mutant^37^ and the Col-0 *hta9-1 hta11-2* double mutant^34^. Unless stated otherwise, all plants were grown in long-days (16 h:8 h light:dark) at 23°C.

### Genomic DNA sequencing using mate-pairs

DNA was extracted from 10–20 seedlings grown under long day conditions, using DNeasy Qiagen kits. About 10 mg of genomic DNA was sonicated to a 4–6 kb size range using the E210 Covaris instrument (Covaris, Inc., USA). Libraries were prepared following Illumina’s protocol (Illumina Mate Pair library kit), starting with size-selected (approximately 5 kb) fragments, which were end-repaired, biotin labeled and circularized. Linear DNA was eliminated by digestion and circularized DNA was fragmented to 300–700 bp using the E210 Covaris. Biotinylated DNA junctions were purified using streptavidin, end-repaired and 3′-adenylated in order to ligate Illumina adapters. Junction fragments were PCR-amplified using Illumina adapter-specific primers and amplified fragments within the 350–650 bp size range were selected for sequencing. Each library was sequenced using 100 base-length read chemistry in a paired-end flow cell on the Illumina GAIIx (2 lanes) or HiSeq2000 (1 lane) (Illumina, USA).

### Mapping and detection of TE insertions using WGS

Reads were mapped with BWA v.0.6.1 using the parameters -R 10000 -l 35 -O 11, and the parameters n 10000 N 10000 -s for sampe, onto the TAIR10 reference sequence. Reads hanging over chromosome ends were removed using picard CleanSam and duplicate pairs were removed using picard MarkDuplicates. TE insertions were detected by implementing TE-Tracker software (available at http://www.genoscope.cns.fr/TE-Tracker)^51^. WGS did not produce enough coverage (<10×) for 16 of the 123 epiRILs analyzed at generation F8, and these 16 epiRILs were not considered further. TE-Tracker is a computational method that we have previously developed for accurately detecting both the identity and destination of newly mobilized TEs in genomes re-sequenced using mate-pair libraries^51^. Importantly, TE-Tracker does not rely on prior annotation, yet is able to integrate it, making the results easily interpretable. Briefly, TE-Tracker uses paired reads mapping information to identify discordant reads that mapped partially over TE-sequences to detect the position of TE insertions. Insertion site positions were refined at the single nucleotide resolution by exploiting sequence information contained in split-reads. To this end, we implemented the software SPLITREADER^12^ (available at https://github.com/LeanQ/SPLITREADER). Homozygous and heterozygous insertions were defined based on the normalized number of reads supporting each insertion event. In addition, this approach enabled us also to identify insertions that were likely present in only one of the 10–20 seedlings used to extract DNA and which reflect transposition during the reproductive phase of the parent. These insertions were also called heterozygous, as this was likely the case and to reflect their very recent ancestry. Conversely, our approach was designed to exclude poorly supported insertions, which could reflect either mapping artifacts or rare somatic events. Finally, visual inspection was carried out for a random sample of over 200 insertion events and their homozygous or heterozygous status was confirmed in each case.

### TE-sequence capture

TE sequence capture was performed on exactly 1000 seedlings in all cases except for the F3 progeny of *hta9 hta11* line 2, where only 477 seedlings were recovered (see main text for details of the plant materials used). Seedlings were grown under control (long-day) conditions and genomic DNA was extracted using the CTAB method^52^. In order to assess the sensitivity of TE-sequence capture, we added 1 ng of genomic DNA extracted from epiRILs 394 (generation F16) to 1 μg of genomic DNA extracted from the 477 F3 seedling of *hta9 hta11* line 2 prior to library construction (i.e., 1:1000 dilution of the spiked-in genomic DNA). Libraries were prepared using 1 μg of DNA and TruSeq paired-end kit (Illumina) following manufacturer instructions. Libraries were then amplified through 7 cycles of ligation-mediated PCR using the KAPA HiFi Hot Start Ready Mix and primers AATGATACGGCGACCACCGAGA and CAAGCAGAAGACGGCATACGAG at a final concentration of 2 µM. 1 µg of multiplexed libraries were then subjected to TE-sequence capture^12^. Enrichment for captured TE sequences was confirmed by qPCR and estimated to be higher than 1000 fold. Pair-end sequencing was performed using one lane of Illumina NextSeq500 and 75 bp reads. About 42 million pairs were sequenced per library and mapped to the TAIR10 reference genome using Bowtie2 v2.3.2^53^ with the arguments –mp 13 –rdg 8,5 –rfg 8,5 –very-sensitive. An improved version of SPLITREADER (available at https://github.com/LeanQ/SPLITREADER) was used to detect new TE insertions. Split-reads, as well as discordant reads mapping partially on the reference sequence of *ATCOPIA93* and *ATCOPIA78* (obtained from RepBase update) were identified, soft clipped and remapped to the TAIR10 reference genome using Bowtie2^53^. Putative insertions supported by at least one split-reads and/or discordant-reads at each side of the insertion sites were retained. Insertions spanning centromeric repeats or coordinates spanning the corresponding donor TE sequence were excluded. In addition, putative TE insertions detected in more than one library were excluded to retain only sample-specific TE insertions. More than 80% of new TE insertions present in epRIL394 F16 were detected, confirming that the sensitivity of our TE-sequence capture and computational approach is higher than 1:1000. In addition, no non-reference insertions were detected in 1000 F1 seedlings of wild-type Col-0, highlighting the specificity of our approach.

### Detection of Tos17 non-reference insertions in rice genomes

A total of 16,784 *Tos17* non-reference flanking sequences^38^ were retrieved from GeneBank and mapped to the reference rice genome using Minimap2 v2.11-r797^54^, which enabled us to identify 14,258 insertion points with high confidence.

### Detection of mutations within transposed copies

Discordant mate-pair reads mapping within a 6 kb interval either upstream or downstream of each insertion site were extracted and re-mapped using Bowtie2^53^ over a library constructed with the specific donor TE sequence only. Sequence variants were detected using samtools mpileup^55^ V1.2.1 and only variants with a quality of at least 30 were kept. Long deletions were detected as regions without coverage and breakpoints were reconstructed by local assembly using Velvet V1.2.09^56^.

### Analysis of global and local enrichment of new TE insertions

To assess if new TE insertions are enriched in specific regions, their number within these regions was compared with that expected from a random distribution. The expected distribution was calculated by randomizing 10^4^ times the position of new TE insertions across the chromosomes (genomic regions showing coverage deviation, the inner pericentromeres, or coordinates spanning the corresponding donor TE sequence were excluded). This set of random positions was used as a control for all subsequent analyses. Insertion distribution over wild-type and *ddm1* intervals was obtained by counting the number of new TE insertions within these, as defined previously^21^. Overrepresentation over genes and neighboring sequences was performed using a meta-gene analysis. Briefly, protein coding gene features were extracted from the TAIR10 annotation and coordinates of non-reference TE insertions with TSDs were crossed with the set of genic features according to the following stepwise hierarchy: 5’ UTR > 3’ UTR > exon > intron > intergenic regions. For insertions that do not overlap protein-coding genes, the distance to the closest gene was calculated and reported as negative or positive distance according to the gene orientation. Overrepresentation over chromatin states was performed by comparing the number of new TE insertions and randomly generated TE insertions associated with each chromatin state^30^. Density of *ATCOPIA93* insertions were obtained by calculating the distance between insertion sites and the middle point of the nearest well positioned nucleosome mapped in Col-0^35^ (Table S5). Gene ontology (GO) analyses were performed using AGRIGO (http://bioinfo.cau.edu.cn/agriGO/) and as input the ID of genes that contain a TE insertion within the limits of their annotation.

### Analysis of chromatin features at insertion sites

Four-kilobase pairs regions centered around insertion sites were defined and used to extract normalized coverage of DnaseI hypersensitivity^57^, Mnase accessibility^58^, H3K27me3^59^, H2A.Z enrichment level, and well-positioned nucleosomes^35^. The same approach was used for the analysis of H2A.Z enrichment in rice^60^ and of htz1 from yeast^61^. Average normalized coverage was then calculated for each bp and plotted using the *smooth.spline* function in R.

### epiQTL mapping of transposition activity

Using TE copy number as phenotype and a total of 126 parental differentially methylated regions (DMRs) that segregate in a Mendelian fashion in the epiRILs (i.e., stable DMRs) as physical markers^21^, we implemented the multiple QTL model (mqmsacn) from the R/qtl package. Genome-wide significance was determined empirically for each trait using 1000 permutations of the data. LOD significance thresholds were chosen to correspond to a genome-wide false positive rate of 5%.

### Modeling of insertion accumulation

In the absence of selection, TE invasion is mostly determined by the rate of transposition and the rate of fixation of insertions by random Mendelian segregation (i.e., genetic drift). Thus, we constructed individual-based transposition-genetic drift models and assuming either that only the reference copy is mobile (master gene model) or that new copies are also mobile (transposon model). As new copies arise in the heterozygous state and because of Mendelian segregation, the number of new copies that can be inherited at the next generation follows a Poisson distribution. We considered three parameters: (i) a fixed rate (*K)* of transposition per copy/per generation, (ii) a fixed rate of excision (*E*) per transposition event/per generation in the case of the two cut-and-paste DNA transposons *VANDAL21* and *ATENSPM3*, and (iii) copy number-dependent concerted silencing of all active copies (*l*), as this type of inhibition has been reported for numerous TEs, including *ATCOPIA93* (*20*). Simulations were run 2500 times using a wide space of parameter values (*K* *=* {0,0.1,…,10}, *E* *=* {0,0.2,…,1} and *l* *=* {20,21,…,100}. Distribution of homozygous and heterozygous insertions between simulated and observed data at F8 was evaluated using a two-dimensional Kolmogorov-Smirnov test (Peacock test), which was specifically designed to assess the goodness-of-fit of mathematical models to observed data^62^ and is implemented in the package Peacock.test in R.

### Transcriptome analysis

RNA from wild-type, *ddm1* epiRIL394 (F8) and epiRIL394 (F16) was isolated using Rneasy Plant Minikit (Qiagen) according to the supplier’s instructions. Contaminating DNA was removed using RQ1 DNase (Promega). 1μg of total RNA was processed using TruSeq Stranded Total RNA kit (Illumina) according to the supplier’s instructions. About 20 M 76nt-long single-end reads were obtained per sample on the Illumina HiSeq2000. Expression level was calculated by mapping reads using STAR v2.5.3a^63^ on the *A. thaliana* reference genome (TAIR10) with the following arguments –outFilterMultimapNmax 50 --outFilterMatchNmin 30 --alignSJoverhangMin 3 --alignIntronMax 10000. Duplicated pairs were removed using picard MarkDuplicates. Counts were normalized and annotations were declared differentially expressed between samples using DESeq2^64^.

### Quantification of expression, copy number, and DNA methylation

Total RNA was extracted using the RNeasy plant mini kit (Qiagen) from plants grown under normal conditions or subjected to heat stress treatment^37^ and cDNA was synthetized using oligo(dT) primers and SuperScript IV reverse transcriptase (ThermoFisher). RT-qPCR results (one biological replicates) are indicated relative to those obtained for *ACT1*. For *ATCOPIA93* copy number and DNA methylation quantification, genomic DNA was extracted from young leaves with the DNeasy Plant Mini kit. McrBC digestion was performed on 400 ng of DNA and qPCR was performed on equal amounts of digested and undigested DNA. Results were expressed as percentage of loss of molecules after McrBC digestion. All qPCR reactions were run on an Applied Biosystems 7500 Real-Time PCR System using LightCycler^®^480 SYBR Green I Master (Roche). Primer sequences are provided in Supplementary Table 1.

### Nectar secretion

Four flowers (stage 14–15, at anthesis) from ten 30-days-old plants of epiRIL394 F9 and F17 were examined for nectar secretion from lateral nectaries after removing the sepals.

### Reporting summary

Further information on research design is available in the Nature Research Reporting Summary linked to this article.

## Supplementary information

Supplementary Information

Peer Review

Reporting Summary

Source Data

## Data Availability

Sequencing data has been deposited in the European Nucleotide Archive (ENA) under project PRJEB5137 and PRJEB29194. A reporting summary for this Article is available as a Supplementary Information file. The datasets generated and analyzed during the current study are available from the corresponding authors upon request. The source data underlying Figs. 1b, 1c, 4b, 4d, 4g, 4h, 5d, and 5e are provided as a Source Data file.

## Code availability

Codes used to detect TE insertions are available at https://github.com/LeanQ/SPLITREADER and http://www.genoscope.cns.fr/TE-Tracker with no restriction to access.

## References

[1] Wicker T (2007). A unified classification system for eukaryotic transposable elements. Nat. Rev. Genet..

[2] Lisch D (2013). How important are transposons for plant evolution?. Nat. Rev. Genet..

[3] Chuong EB, Elde NC, Feschotte C (2017). Regulatory activities of transposable elements: From conflicts to benefits. Nat. Rev. Genet..

[4] Huang CRL, Burns KH, Boeke JD (2012). Active transposition in genomes. Annu. Rev. Genet..

[5] Kidwell MG, Lisch D (1997). Transposable elements as sources of variation in animals. Proc. Natl Acad. Sci. USA.

[6] Chuong EB, Elde NC, Feschotte C (2016). Regulatory evolution of innate immunity through co-option of endogenous retroviruses. Science.

[7] Jangam D, Feschotte C, Betrán E (2018). Transposable element domestication as an adaptation to evolutionary conflicts. Trends Genet..

[8] Sultana T, Zamborlini A, Cristofari G, Lesage P (2017). Integration site selection by retroviruses and transposable elements in eukaryotes. Nat. Rev. Genet..

[9] Slotkin RK, Martienssen R (2007). Transposable elements and the epigenetic regulation of the genome. Nat. Rev. Genet..

[10] Ossowski S (2010). The rate and molecular spectrum of spontaneous mutations in Arabidopsis thaliana. Science.

[11] Weng Mao-Lun, Becker Claude, Hildebrandt Julia, Neumann Manuela, Rutter Matthew T., Shaw Ruth G., Weigel Detlef, Fenster Charles B. (2018). Fine-Grained Analysis of Spontaneous Mutation Spectrum and Frequency in Arabidopsis thaliana. Genetics.

[12] Quadrana L (2016). The Arabidopsis thaliana mobilome and its impact at the species level. Elife.

[13] Johannes F (2009). Assessing the impact of transgenerational epigenetic variation on complex traits. Plos Genet..

[14] Lippman Z (2004). Role of transposable elements in heterochromatin and epigenetic control. Nature.

[15] Vongs A, Kakutani T, Martienssen RA, Richards EJ (1993). Arabidopsis thaliana DNA methylation mutants. Science.

[16] Zhu YO, Siegal ML, Hall DW, Petrov DA (2014). Precise estimates of mutation rate and spectrum in yeast. Proc. Natl Acad. Sci..

[17] Denver DR (2009). A genome-wide view of *Caenorhabditis elegans* base-substitution mutation processes. Proc. Natl Acad. Sci..

[18] Keightley PD (2009). Analysis of the genome sequences of three *Drosophila melanogaster* spontaneous mutation accumulation lines. Genome Res..

[19] Mirouze M (2009). Selective epigenetic control of retrotransposition in Arabidopsis. Nature.

[20] Marí-Ordóñez A (2013). Reconstructing de novo silencing of an active plant retrotransposon. Nat. Genet..

[21] Cortijo S (2014). Mapping the epigenetic basis of complex traits. Science.

[22] Colomé-Tatché M (2012). Features of the Arabidopsis recombination landscape resulting from the combined loss of sequence variation and DNA methylation. Proc. Natl Acad. Sci..

[23] Deininger PL, Batzer MA, Hutchison CA, Edgell MH (1992). Master genes in mammalian repetitive DNA amplification. Trends Genet..

[24] Drake JW, Charlesworth B, Charlesworth D, Crow JF (1998). Rates of spontaneous mutation. Genetics.

[25] Engels WR, Johnson-Schlitz DM, Eggleston WB, Sved J (1990). High-frequency P element loss in Drosophila is homolog dependent. Cell.

[26] Doseff A, Martienssen R, Sundaresan V (1990). Somatic excision of the *Mu1* transposable element of maize. Nucleic Acids Res. Acids Res..

[27] Soppe WJJ (2002). DNA methylation controls histone H3 lysine 9 methylation and heterochromatin assembly in Arabidopsis. EMBO J..

[28] Ito T (2015). Genome-wide negative feedback drives transgenerational DNA methylation dynamics in Arabidopsis. PLoS Genet..

[29] Lloyd JP, Seddon AE, Moghe GD, Simenc MC, Shiu S-H (2015). Characteristics of plant essential genes allow for within- and between-species prediction of lethal mutant phenotypes. Plant Cell.

[30] Sequeira-Mendes J (2014). The functional topography of the Arabidopsis genome is organized in a reduced number of linear motifs of chromatin states. Plant Cell.

[31] Osakabe A (2018). Histone H2A variants confer specific properties to nucleosomes and impact on chromatin accessibility. Nucleic Acids Res.

[32] Fu Y (2013). Mobilization of a plant transposon by expression of the transposon-encoded anti-silencing factor. EMBO J..

[33] Zilberman, D., Coleman-Derr, D., Ballinger, T. & Henikoff, S. Histone H2A.Z and DNA methylation are mutually antagonistic chromatin marks. *Nature* **456**, 125–129 (2008). 10.1038/nature07324 PMC2877514 18815594

[34] March-Díaz R (2008). Histone H2A.Z and homologues of components of the SWR1 complex are required to control immunity in Arabidopsis. Plant J..

[35] Lyons DB, Zilberman D (2017). DDM1 and lsh remodelers allow methylation of DNA wrapped in nucleosomes. Elife.

[36] Zlatanova J, Thakar A (2008). H2A.Z: View from the Top. Structure.

[37] Ito H (2011). An siRNA pathway prevents transgenerational retrotransposition in plants subjected to stress. Nature.

[38] Miyao A (2003). Target site specificity of the Tos17 retrotransposon shows a preference for insertion withn genes and against insertion in retrotransposon-rich regions of the genome. Plant Cell.

[39] Mularoni L (2012). Retrotransposon Ty1 integration targets specifically positioned asymmetric nucleosomal DNA segments in tRNA hotspots. Genome Res..

[40] Coleman-Derr D, Zilberman D (2012). Deposition of histone variant H2A.Z within gene bodies regulates responsive genes. PLoS Genet..

[41] Bhatia V, Maisnam J, Jain A, Sharma KK, Bhattacharya R (2015). Aphid-repellent pheromone E-β-farnesene is generated in transgenic Arabidopsis thaliana over-expressing farnesyl diphosphate synthase2. Ann. Bot..

[42] Lin IW (2014). Nectar secretion requires sucrose phosphate synthases and the sugar transporter SWEET9. Nature.

[43] Hoffmann MH (2003). Flower visitors in a natural population of Arabidopsis thaliana. Plant Biol..

[44] Li P (2014). Multiple *FLC* haplotypes defined by independent cis-regulatory variation underpin life history diversity in Arabidopsis thaliana. Genes Dev..

[45] Coustham V (2012). Quantitative modulation of polycomb silencing underlies natural variation in vernalization. Science.

[46] Berry S, Dean C (2015). Environmental perception and epigenetic memory: mechanistic insight through *FLC*. Plant J..

[47] Kawakatsu T (2016). Epigenomic diversity in a global collection of Arabidopsis thaliana accessions. Cell.

[48] Richter TE, Ronald PC (2000). The evolution of disease resistance genes. Plant Mol. Biol..

[49] Yelagandula R (2014). The histone variant H2A.W defines heterochromatin and promotes chromatin condensation in Arabidopsis. Cell.

[50] Tsukahara S (2012). Centromere-targeted de novo integrations of an LTR retrotransposon of *Arabidopsis lyrata*. Genes Dev..

[51] Gilly A (2014). TE-Tracker: Systematic identification of transposition events through whole-genome resequencing. BMC Bioinforma..

[52] Murray G, Thompson WF (1980). Rapid isolation of high molecular weight plant DNA. Nucleic Acids Res..

[53] Langmead B, Salzberg SL (2012). Fast gapped-read alignment with Bowtie 2. Nat. Methods.

[54] Li H (2018). Minimap2: pairwise alignment for nucleotide sequences. Bioinformatics.

[55] Li H (2009). The sequence alignment/map format and SAMtools. Bioinformatics.

[56] Zerbino DR, Birney E (2008). Velvet: Algorithms for de novo short read assembly using de Bruijn graphs. Genome Res..

[57] Sullivan AM (2014). Mapping and dynamics of regulatory DNA and transcription factor networks in A. thaliana. Cell Rep..

[58] Li G (2014). ISWI proteins participate in the genome-wide nucleosome distribution in Arabidopsis. Plant J..

[59] Li C (2015). The Arabidopsis SWI2/SNF2 chromatin remodeler BRAHMA regulates polycomb function during vegetative development and directly activates the flowering repressor gene *SVP*. PLoS Genet..

[60] Zahraeifard S (2018). Rice H2A.Z negatively regulates genes responsive to nutrient starvation but promotes expression of key housekeeping genes. J. Exp. Bot..

[61] Gu M, Naiyachit Y, Wood TJ, Millar CB (2015). H2A.Z marks antisense promoters and has positive effects on antisense transcript levels in budding yeast. BMC Genom..

[62] Peacock JA (1983). Two-dimensional goodness-of-fit testing in astronomy. Mon. Not. R. Astron. Soc..

[63] Dobin A (2013). STAR: ultrafast universal RNA-seq aligner. Bioinformatics.

[64] Love MI, Huber W, Anders S (2014). Moderated estimation of fold change and dispersion for RNA-seq data with DESeq2. Genome Biol..

