# Supplementary material for: Transposition favors the generation of large effect mutations that may facilitate rapid adaption
Source: Nat Commun. 2019 Jul 31;10:3421. doi: 10.1038/s41467-019-11385-5 (PMC6668482; doi:10.1038/s41467-019-11385-5)
Supplement: Supplementary file 1 — Supplementary Information [file 41467_2019_11385_MOESM1_ESM.pdf]

Transposition favors the generation of large effect mutations that may facilitate rapid adaption

Quadrana *et al.*

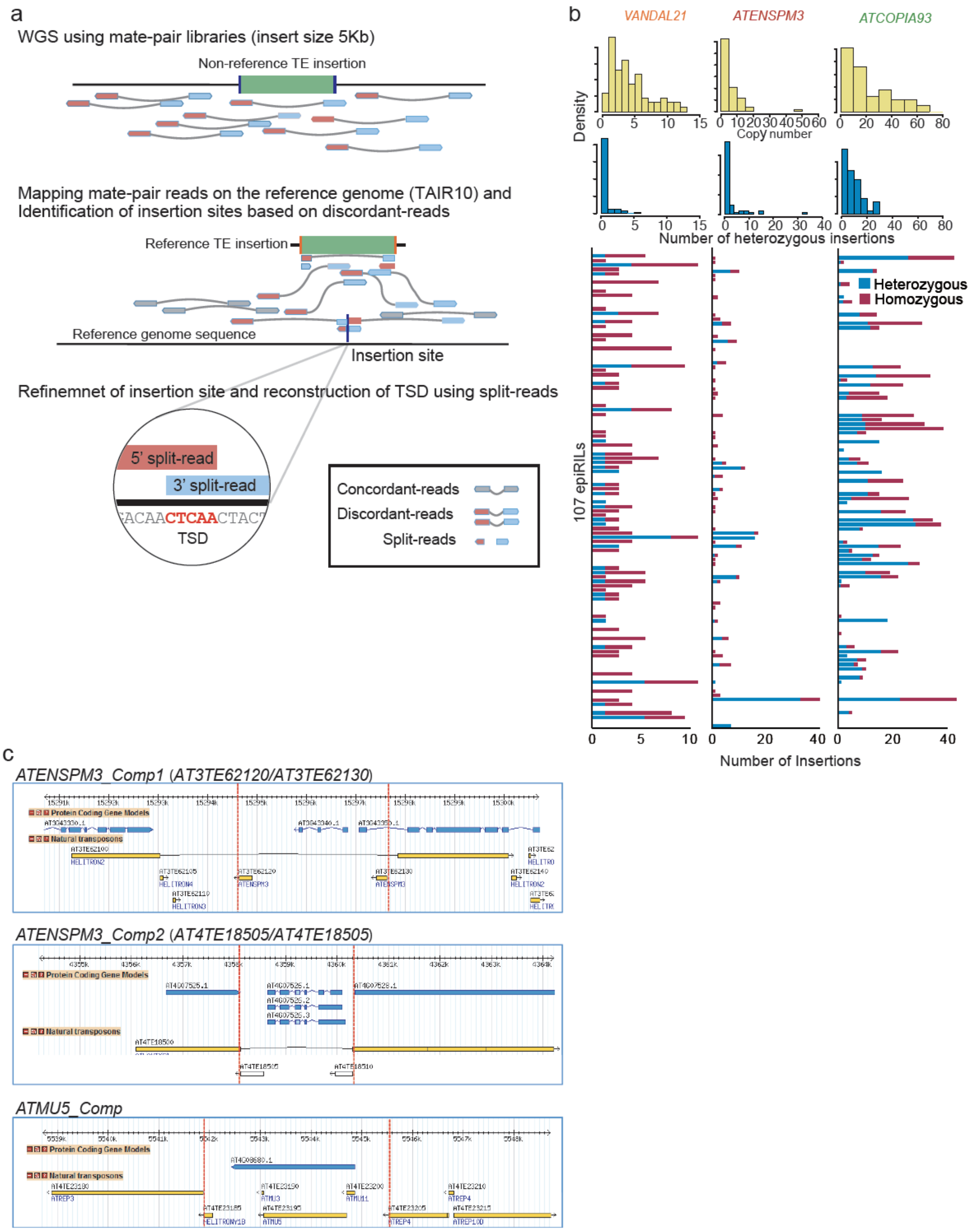

Supplementary Fig. 1. Identification of new TE insertions in epiRILs. a. Schematic representation of the bioinformatic method used to detect new TE insertions. Sequenced reads around a newly inserted TE-copy (top half) produce discordant read mappings when aligned with the reference sequence (bottom half). Dashed light-red and light-blue arrows represent the mate-pairs reads linking the left and right extremities of the insertion breakpoint with the donor TE sequence. **b.** The top two rows of panels indicate the distribution of the number of private insertions (top: all; bottom: heterozygous only) among epiRILs for each TE family. The number of homozygous and heterozygous private insertions in each epiRIL (ordered by name) is indicated below for each of the three TE families. **c**. Genome browser view of the three composite mobile TEs (delineated by the dotted red lines) identified in the present study.

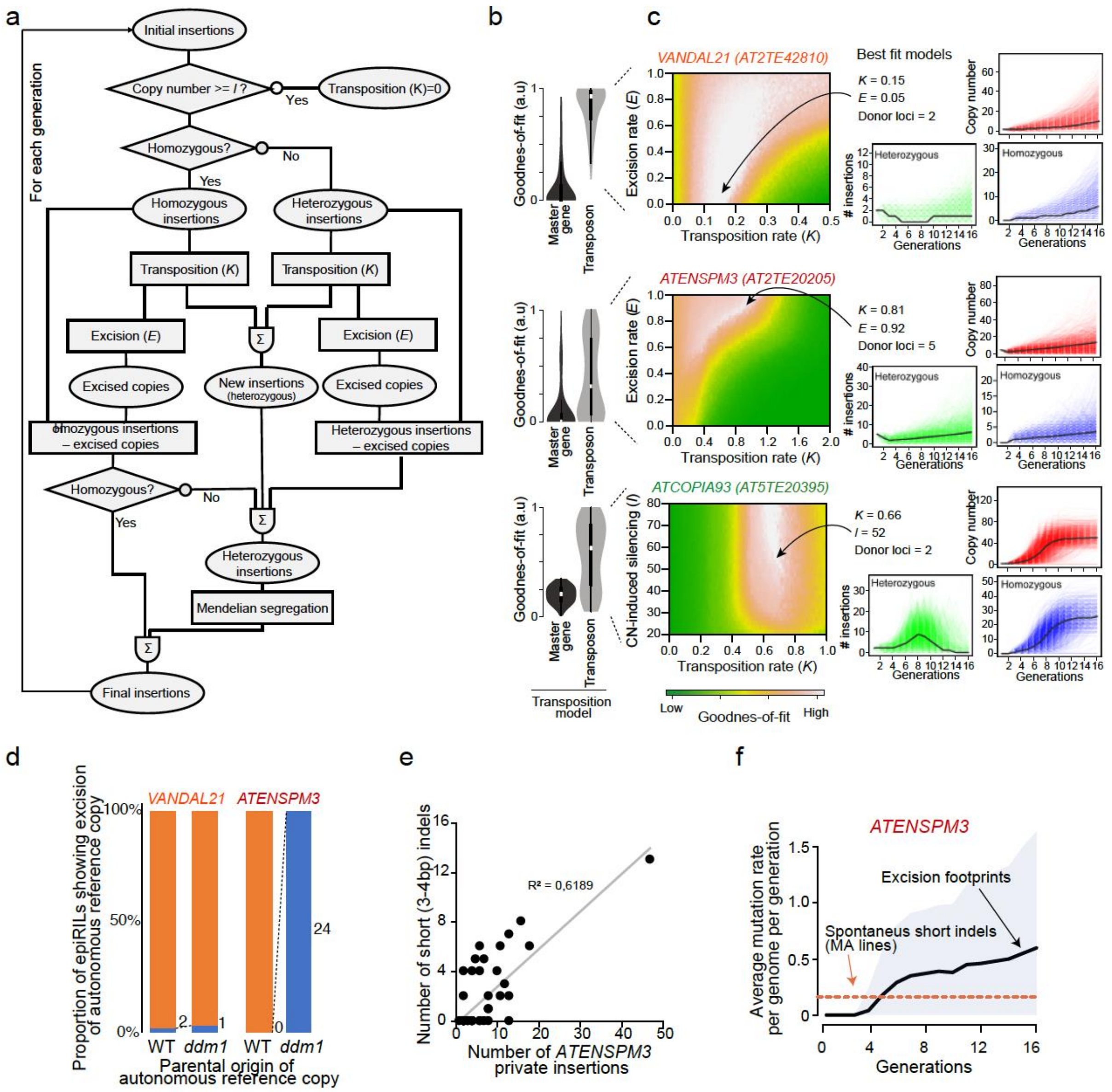

Supplementary Fig. 2. Modeling of insertion accumulation dynamics. **a**. Schematic representation of the transposition-drift model developed to reconstruct transposition dynamics. **b**. Goodness-of-fit between observed data simulated data obtained under a "master gene" or "transposon" model. **c**. Goodness-of-fit between observed and simulated data obtained under a "transposon" model and with different parameters values. Patterns of insertion accumulation produced by the best fitted models are shown. **d**. Proportion and number of epiRILs showing excision of the autonomous reference copy for *VANDAL21* and *ATENSPM3* in relation to their parental origin. **e**. Correlation between the number of private *ATENSPM3* insertions and excision footprints detected in the epiRILs. **f**. Average indel rate across generations contributed by *ATENSPM3* mobilization (black line) and spontaneous mutations (red line) obtained in the epiRILs and MA lines[10] (bottom), respectively. Grey area represents 95% C.I.

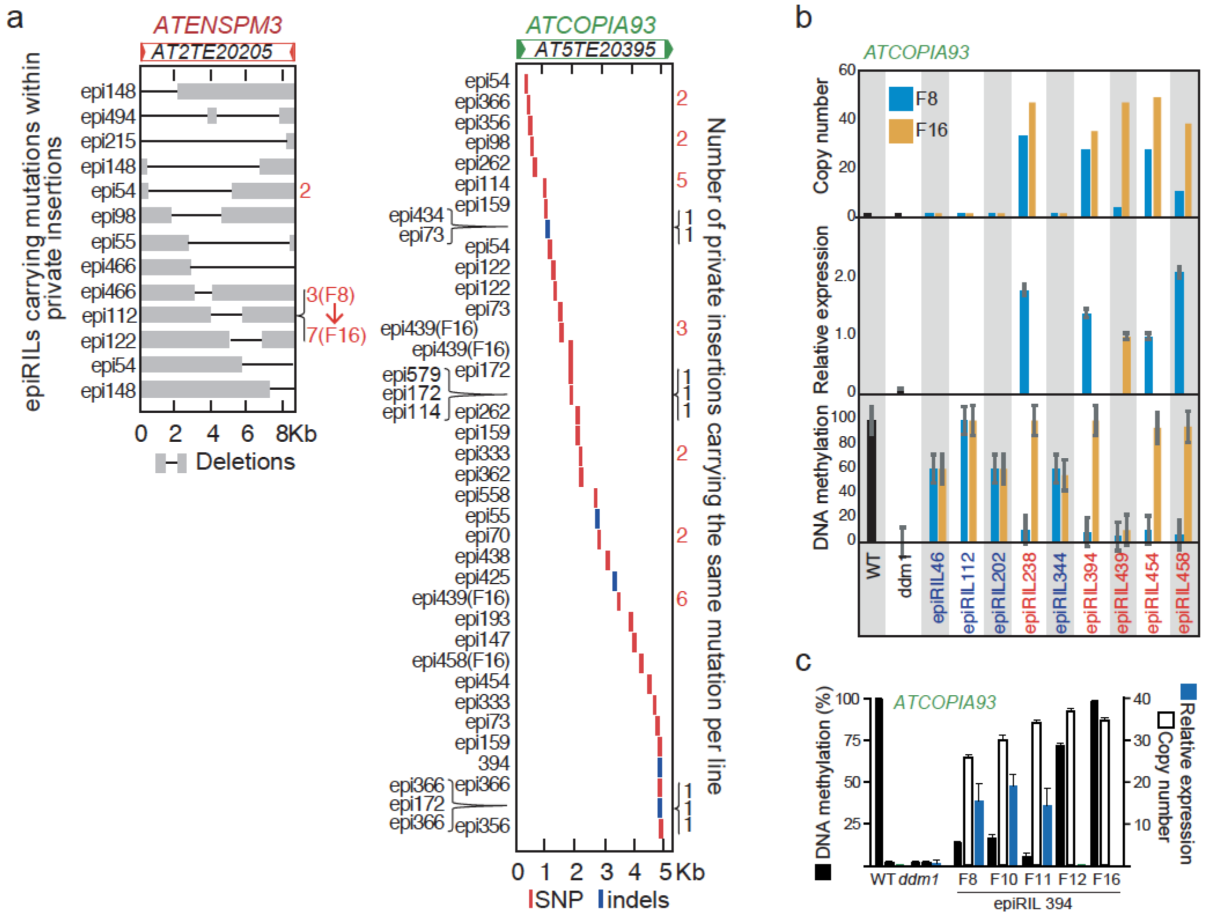

Supplementary Fig. 3. Transposition chain reaction and silencing of *ATCOPIA93* at later generations. a. Mutations detected within private insertions of *ATENSPM3* and *ATCOPIA93*. Carrier epiRILs as well as the number of private insertions with the mutation are indicated in each case on the left and right side of each panel, respectively. **b.** *ATCOPIA93* copy number as well as q-PCR analyses of DNA methylation and expression of *ATCOPIA93* in wild-type, *ddm1* and nine epiRILs at F8 and F16. Data are mean ± s.d. (n = 2 independent biological experiments). Samples containing or lacking TE insertions are highlighted in red and blue, respectively. **c**. q-PCR analyses of DNA methylation, copy number and expression of *ATCOPIA93* in wild type, *ddm1* and epiRIL394, taken at F8 and more advanced generations. Data are mean ± s.d. (n = 1 independent biological experiment including at least ten seedlings)

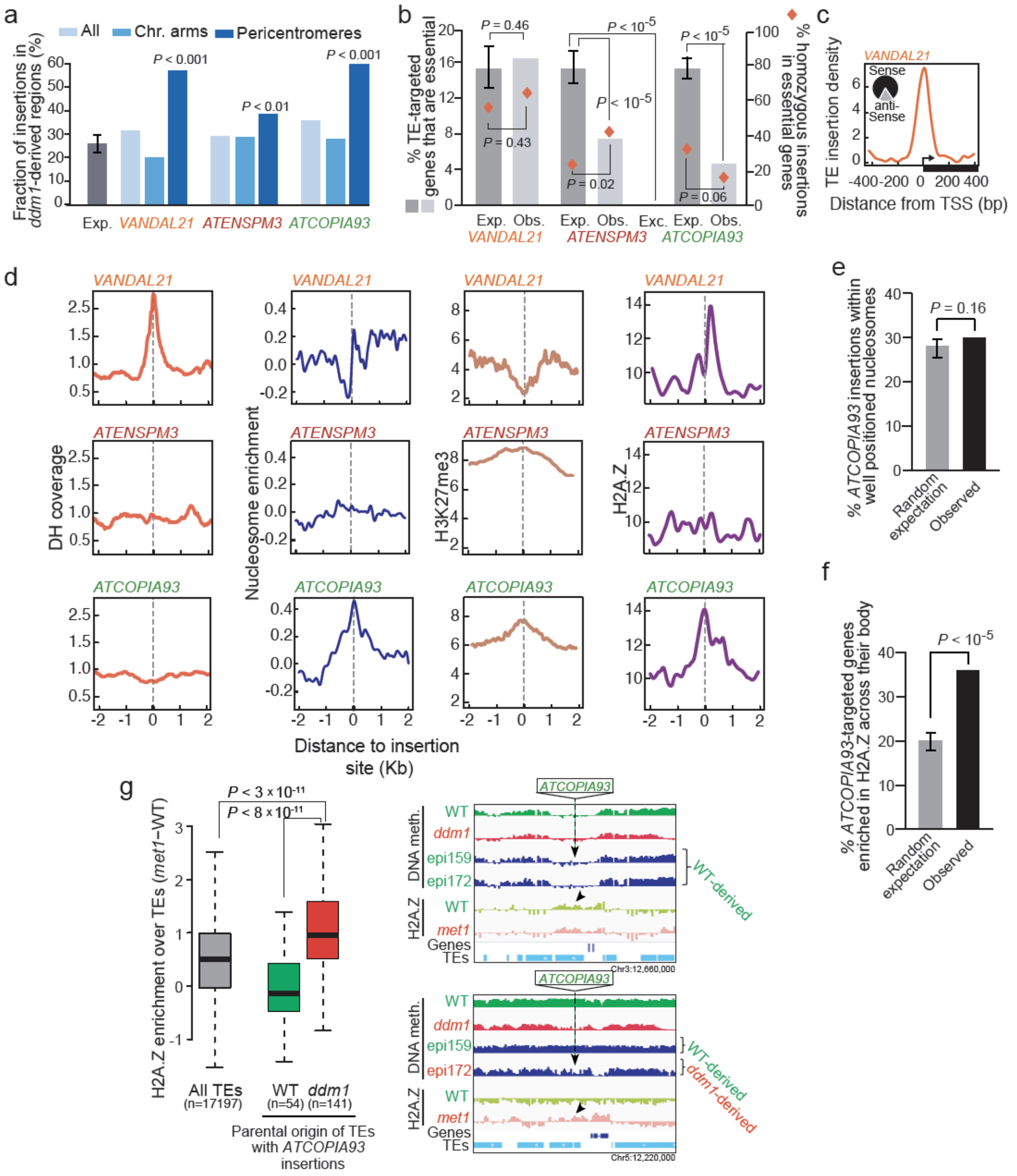

Supplementary Fig. 4. TEs exhibit strong and diverse chromatin-associated insertion biases towards genes. a. Proportion of private TE insertions located in *ddm1* intervals for the two compartments considered, chromosome arms and perictentromeric regions. Statistical significance between expected and observed data was obtained using the Chi-square test. **b.** Fraction of essential genes containing *VANDAL21*, *ATENSPM3* or *ATCOPIA93* insertions as well as short indels compatible with *ATENSPM3* excision footprints in the epiRILs. the proportion of homozygous insertions is also indicated. Statistical significance for each comparison was obtained using the Chi-square test. **c.** Density of *VANDAL21* insertions around transcriptional start sites (TSS). The fraction of insertions that are in the same (sense) or opposite (antisense) orientation relative to the targeted gene are indicated. **d**. Meta-analysis of of DNAse hypersensitivity (DH), nucleosome, H3K7me3 and H2A.Z levels around insertion sites for *VANDAL21*, *ATENSPM3* and *ATCOPIA93*. **e**. Proportion of *ATCOPIA93* private insertions within well-positioned nucleosomes. Expected distribution was obtained by randomizing 1000 times insertion site positions across the genome and performing a randomization test. Errors bars represent the 95% confidence interval. **f**. Fraction of genes containing *ATCOPIA93* private insertions that are also enriched in H2A.Z across their body. Expected distribution was obtained by randomizing 1000 times insertion site positions across the genome and performing a randomization test. Errors bars represent the 95% confidence interval. **g**. Relative level of H2A.Z in *met1* compared to wild-type over all TEs (grey box) and in the subset of TEs that contain

ATCOPIA93 insertions in the epiRILs. For each boxplot, the lower and upper bounds of the box indicate the first and third quartiles, respectively, and the center line indicates the median. Genome browser views of DNA methylation and H2A.Z over TEs containing *ATCOPIA93* insertions within wild-type and *ddm1* intervals (top and bottom panel, respectively) are depicted on the right. Insertion sites are indicated by an arrow and samples showing hypomethylation across the region are highlighted in red. Statistical significance for each comparison were obtained by Mann–Whitney test.

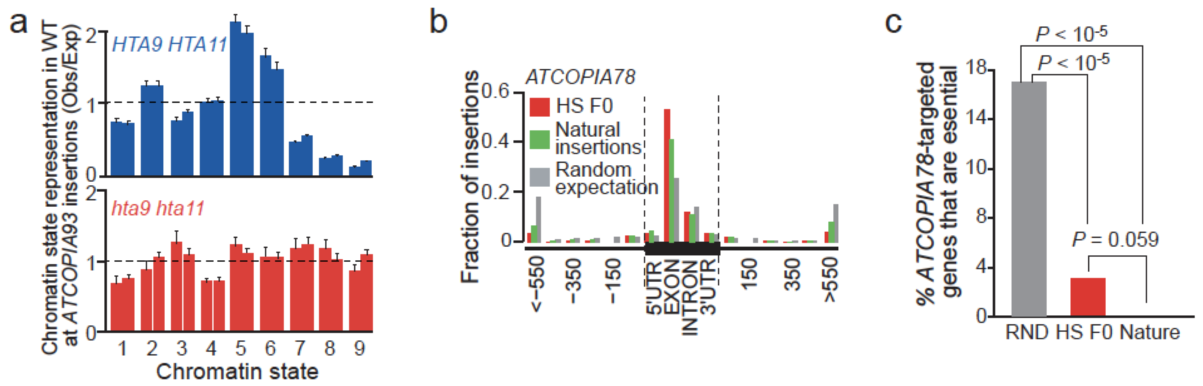

Supplementary Fig. 5. H2A.Z guides the integration of *Ty1/Copia* retrotransposons. a. Observed/expected ratio (O/E) of insertion sites in relation to the nine chromatin states defined in *A. thaliana*. Error bars represent the 95% confidence interval obtained by 1000 boots-traps. **b**. Metagene analysis showing the distribution of new *ATCOPIA78* insertions detected in heat-stressed *nrpd1* mutant plants (HS F0) or in natural population of *A. thaliana* (Natural insertions). UTR, untranslated transcribed region. **c**. Fraction of essential genes containing experimentally induced (HS F0) or natural *ATCOPIA78* insertions. Statistical significance for each comparison was obtained using the Chi-square test.

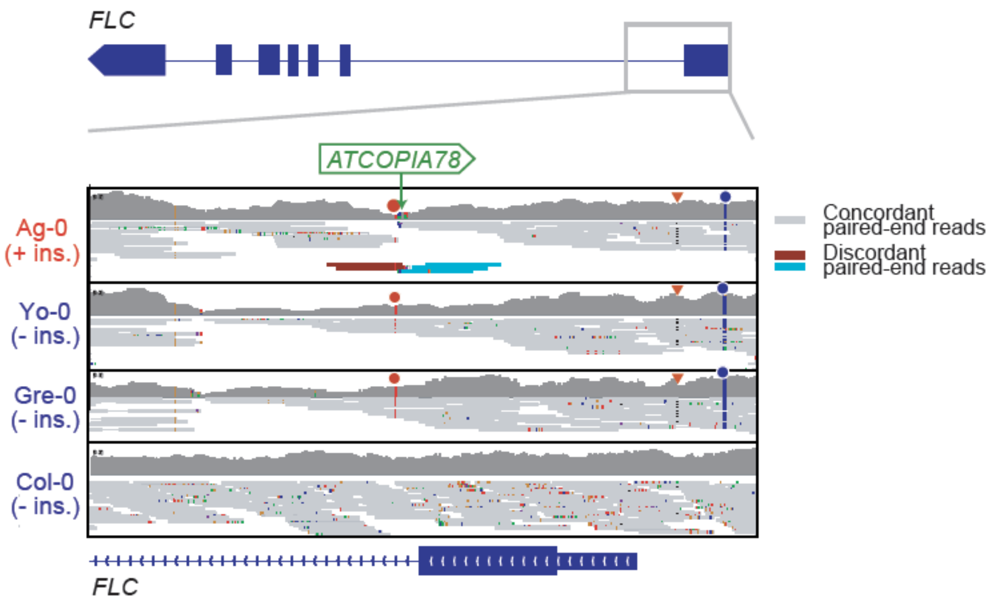

Supplementary Fig. 6. Ag-0 contains a recent *ATCOPIA78* insertion in the first intron of *FLC*. Genome browser view of sequence reads produced by WGS over *FLC* for the accessions Ag-0, Yo-0, Gre-0 and Col-0. Concordant and discordant paired-reads, as well as the sense or antisense orientation for the latter, are indicated in grey, brown and cyan, respectively. The presence of common polymorphisms associated with vernalization response are indicated by the small circles and the arrowhead

Supplementary Table 1

Primer_name	Sequence (5'-3')
ACT_For	CTAAGCTCTCAAGATCAAAGGC
ACT_Rev	AACATTGCAAAGAGTTTCAAGG
ATCOPIA93_For	GATAGAGGAGATAGAAGATCTACAACTGG
ATCOPIA93_Rev	CTCTATACTCCGATTCTGCACTCGAACA
AT5G36220-3met-For	CCGAACACTTCACCAGATCA
AT5G36220-3met -Rev	CAGACCCGGGTAACTTTTGA
FLC_ex1_ex5_F	CATCCGTCGCTCTTCTCGTC
FLC_ex1_ex5_R	TCTAGTCACGGAGAGGGCAG
