# Supplementary material for: Transposition favors the generation of large effect mutations that may facilitate rapid adaption
Source: Nat Commun. 2019 Jul 31;10:3421. doi: 10.1038/s41467-019-11385-5 (PMC6668482; doi:10.1038/s41467-019-11385-5)
Supplement: Supplementary file 3 — Reporting Summary [file 41467_2019_11385_MOESM3_ESM.pdf]

nature research

Corresponding author(s): Leandro Quadrana, Vincent Colot

Last updated by author(s): Jul 10, 2019

Reporting Summary

Nature Research wishes to improve the reproducibility of the work that we publish. This form provides structure for consistency and transparency in reporting. For further information on Nature Research policies, see Authors & Referees and the Editorial Policy Checklist.

Statistics

For all statistical analyses, confirm that the following items are present in the figure legend, table legend, main text, or Methods section.

n/a	Confirmed	
☐	☒	The exact sample size (*n*) for each experimental group/condition, given as a discrete number and unit of measurement
☐	☒	A statement on whether measurements were taken from distinct samples or whether the same sample was measured repeatedly
☐	☒	The statistical test(s) used AND whether they are one- or two-sided<br>*Only common tests should be described solely by name; describe more complex techniques in the Methods section.*
☒	☐	A description of all covariates tested
☐	☒	A description of any assumptions or corrections, such as tests of normality and adjustment for multiple comparisons
☐	☒	A full description of the statistical parameters including central tendency (e.g. means) or other basic estimates (e.g. regression coefficient) AND variation (e.g. standard deviation) or associated estimates of uncertainty (e.g. confidence intervals)
☐	☒	For null hypothesis testing, the test statistic (e.g. *F*, *t*, *r*) with confidence intervals, effect sizes, degrees of freedom and *P* value noted<br>*Give P values as exact values whenever suitable.*
☒	☐	For Bayesian analysis, information on the choice of priors and Markov chain Monte Carlo settings
☒	☐	For hierarchical and complex designs, identification of the appropriate level for tests and full reporting of outcomes
☒	☐	Estimates of effect sizes (e.g. Cohen's *d*, Pearson's *r*), indicating how they were calculated

Our web collection on statistics for biologists contains articles on many of the points above.

Software and code

Policy information about availability of computer code

Data collection	NA
Data analysis	TE-Tracker (Gilly et al., 2014): available at http://www.genoscope.cns.fr/TE-Tracker<br>SPLITREADER (Quadrana et al., 2016): available at https://github.com/LeanQ/SPLITREADER<br>Bowtie2 v2.3.2 (Langmead and Salzberg, 2012): available at https://sourceforge.net/projects/bowtie-bio/files/bowtie2/2.3.5.1/<br>Minimap2 v2.11-r797 (Li, 2018): available at https://github.com/lh3/minimap2<br>Velvet V1.2.09 (Zerbino and Birney, 2008): available at https://www.ebi.ac.uk/~zerbino/velvet/<br>STAR v2.5.3a (Dobin et al., 2013): available at https://github.com/alexdobin/STAR<br>DESeq2 (Love et al., 2014): available at https://bioconductor.org/packages/release/bioc/html/DESeq2.html<br>BWA v.0.6.1 (available at https://sourceforge.net/projects/bio-bwa/files/)<br>Picard Tools (available at https://broadinstitute.github.io/picard/)<br>samtools V1.2.1 (Li et al., 2009): Available at http://www.htslib.org/download/

For manuscripts utilizing custom algorithms or software that are central to the research but not yet described in published literature, software must be made available to editors/reviewers. We strongly encourage code deposition in a community repository (e.g. GitHub). See the Nature Research guidelines for submitting code & software for further information.

Data

Policy information about availability of data

All manuscripts must include a data availability statement. This statement should provide the following information, where applicable:

- Accession codes, unique identifiers, or web links for publicly available datasets
- A list of figures that have associated raw data
- A description of any restrictions on data availability

Sequencing data has been deposited in the European Nucleotide Archive (ENA) under project PRJEB5137 and PRJEB29194.

Field-specific reporting

Please select the one below that is the best fit for your research. If you are not sure, read the appropriate sections before making your selection.

☒ Life sciences ☐ Behavioural & social sciences ☐ Ecological, evolutionary & environmental sciences

For a reference copy of the document with all sections, see nature.com/documents/nr-reporting-summary-flat.pdf

Life sciences study design

All studies must disclose on these points even when the disclosure is negative.

Sample size	We sequenced 123 epiRILs at generation F8, 10 epiRILs at generation F16 and siblings of the two founder Col-0 and ddm1 parents. In addition, we performed TE sequence capture followed by Illumina sequencing on exactly 1000 F3 seedlings plants of two lines derived from the cross between epi54 X Col-0 and between epi54 x hta9-11, F1 plants of nrpe1 plants subjected to control or heat stress treatments. In the case of hta9 hta11 line 2, only 477 seedlings were recovered and pooled to perform TE-sequence capture.
Data exclusions	WGS did not produce sufficient coverage (<10X) for 16 of the 123 epiRILs analyzed at generation F8, and these 16 epiRILs were not considered further (see Table S1)
Replication	The epiRIL population, by design, consist in 123 independent lines representing replicated experiments. For TE-sequence capture experiments, two independent lines were obtained and sequenced. Experiments with natural accessions were performed using 10 individuals in each case.
Randomization	The 123 epiRILs used for methylome analysis (Colome-tatche et al, 2012) and whole genome sequencing (this study) were randomly selected.
Blinding	The investigators were not blinded to group allocation during data collection and analyses

Reporting for specific materials, systems and methods

We require information from authors about some types of materials, experimental systems and methods used in many studies. Here, indicate whether each material, system or method listed is relevant to your study. If you are not sure if a list item applies to your research, read the appropriate section before selecting a response.

Materials & experimental systems

n/a	Involved in the study	
☒	☐	Antibodies
☒	☐	Eukaryotic cell lines
☒	☐	Palaeontology
☒	☐	Animals and other organisms
☒	☐	Human research participants
☒	☐	Clinical data

Methods

n/a	Involved in the study	
☒	☐	ChIP-seq
☒	☐	Flow cytometry
☒	☐	MRI-based neuroimaging
