# Supplementary material for: Transposition favors the generation of large effect mutations that may facilitate rapid adaption
Source: Nat Commun. 2019 Jul 31;10:3421. doi: 10.1038/s41467-019-11385-5 (PMC6668482; doi:10.1038/s41467-019-11385-5)
Supplement: Supplementary file 2 — Peer Review [file 41467_2019_11385_MOESM2_ESM.pdf]

Reviewers' comments:

Reviewer #1 (Remarks to the Author):

This is well-executed study of how active transpsons behave en planta, and provides insight into how transposon excision/insertion can impact gene function and trait evolution. Using the EpiRIL system developed by the Colot lab, transposon properties are experimentally tested and modeled, including which transposons are active (three dominant transposon families), excision and transposition rates, insertion preference relative to gene and epigenetic features, and the natural limits on transposon accumulation by epigenetic silencing. One strength of this manuscript is that in addition to identifying correlative relationships, such as the preferential insertion of Ty1/copia at H2A.Z sites, they also experimentally confirm the observation, in this case by crossing the appropriate EpiRIL with and H2A.Z altered mutant (hta9 hta1 mutant). Additionally, the last section discussing the impact of ATCOPIA78/93 on gene expression and the example of the Ag-0 FLC/ATCOPIA78 helps to show that the synthetic EpiRIL system can provide insight into what happens in wild populations.

The only major criticisms I have is that they should provide more of an introduction to the EpiRIL system, in particular the fact that each EpiRIL has a mosaic genome composed of the ddm1 and wt intervals. It is mentioned when relevant to specific analysis, but would be helpful to have it laid out more explicitly as this is an important property of the EpiRILs that distinguishes them from a wild type plant. As part of this, I think it would be helpful to have some analysis of the rate of insertion of the three major transposons into the wild type and ddm1 intervals. The Fig S4f does describe this for ATCOPIA93, but I think it would be helpful to do so explicitly and earlier as it was a question I had while reading through the manuscript. I doubt that preferential "epi-type" insertion would confound any of the major conclusions of the manuscript, but I would like to these discussed more directly with some basic analysis.

Smaller issues:
Fig S5c - The description of S5c seems to be missing from the figure caption.

113 (Figure S2B). Different format than other places.

208-211 "As a result, there were almost three times as many essential genes targeted by ATCOPIA93 in mutant than in wild type seedlings (Fig. 4b). These results indicate that by targeting H2A.Z-containing nucleosome arrays, ATCOPIA93 avoids essential genes and thus minimizes the risks of being lost together with its host."
The observed lower rate of targeting of essential genes in wild type is a really interesting observation, but I don't quite get the proposed mechanism as it relates to H2A.Z. Does the lower H2A.Z levels in the mutants result in more distributed insertion events thus increasing the essential gene targeting? Do essential genes have higher H2A.Z in the mutant and thus higher targeting? I think a sentence or two to describe exactly what the authors think is happening here would be helpful.

137-47 - This is a really interesting section, but I'm wondering if they could more extensively test the original siRNA/Tn-load hypothesis described in Mari-Ordonez et al. That is, if they did a finer grain analysis of copy number and Tn silencing in EpiRIL458 between the F8-F16 (Fig S3b) it would help clarify if there is a specific copy number range that triggers silencing of all targets as described in Mari-Ordonez et al., or is silencing more gradually acquired across a few generations. This isn't critical to the current study, but would provide strong, independent confirmation of a very interesting and important model of transposon silencing.

Reviewer #2 (Remarks to the Author):

Review of "Transposition generates high rates of targeted mutations that fuel rapid adaptive changes"

by L. Quadrana et al., NSCOMMS-19-13366.

This paper reports the results of a mutation accumulation experiment in A. thaliana, in which mutations were induced by an epigenetic induction of transposable element activity. The authors observed that (i) new TE insertions tend to accumulate in their experimental lines, (ii) this accumulation was exponential, supporting the idea that the newly generated insertions were themselves able to transpose, (iii) TE insertions were not uniformly distributed in the genome, but appeared to be more frequent around genes, (iv) different TE families display different insertion distribution patterns related to different chromatin signatures, (v) in particular, Ty1/copia retrotransposons were associated with a specific histone variant, H2A.Z, which is a marker of environmentally-induced genes.

Although each of these results is not totally unheard from the literature, the size of the experiment, the precision of the molecular analysis, and the accumulation of consistent evidence clearly justifies the publication of this study in a first-rank journal. As far as I know, this is the best TE-induced mutation accumulation study in any organism, and the authors' endeavor at providing a deep and detailed analysis of each result is remarkable (at the cost of overcrowded stamp-sized figures). As a non-specialist in epigenetics and chromatin states, I will focus my review on population genetics considerations, which should not be interpreted as a lack of interest for the main results of this study.

On the down side, I found the "adaptation" part of the manuscript much less robust than all other claims, and I am not convinced that this deserves so much emphasis (one third of the title), given the weakness of empirical evidence. If I interpret correctly the results, the authors have shown that in one epiRIL selected for its richness in TE insertions in plastic genes, TEs tend to inhibit gene expression at generation F8, but not at generation F17. The authors further showed that this change in expression is associated with the expected phenotypic consequences for one gene. Independently, the authors noticed that a preexisting Copia insertion in the FLC gene was polymorphic in A. thaliana, and that this insertion was associated with a change in gene expression in specific environmental conditions (heat stress). The authors' hypothesis is that this insertion could be present in some populations due to local adaptation, which is difficult to assess without additional evidence. From these two observations ((i) in at least one epiRIL, some TE insertions located in plastic genes can decrease temporarily gene expression, and (ii) there is a natural TE insertion that could be associated with local adaptation), I do not think it is fair to conclude that "targeted mutations fuel rapid adaptive changes". The idea that there might be an adaptive process associated with the chromatin state TE insertion preference is a hypothesis proposed by the authors as a possible interpretation for their results, and should not be presented as a result of the study, which is about targeted insertions. In my opinion, this hypothesis as such is rather vague, and seems to stem into an adaptationist program rather than in modern population genetics (adaptive "mutator" loci are highly unlikely in recombining organisms). In particular, some of the claims related to the adaptive potential of the reported mechanisms are vague and remain unchallenged throughout the manuscript. For instance, line 62, "endows TEs with a unique ability to accelerate the generation of adaptive mutations while minimizing the mutational load" does not seem to rely on any direct evidence. In the same way, the discussion paragraph lines 288 - 294 states that "retrotransposons have been turned into epigenetically and environmentally sensitive engines of adaptive innovation", and that "such an evolutionary scenario provides a plausible explanation for the invasive success of this superfamily". A necessary condition for natural selection to favor Copia invasion is that the effects of TE insertions are in average more beneficial than deleterious, which I do not really find "plausible". In any case, testing adaptation as a consequence and/or a cause of TE specific insertion patterns is, as stated by the authors themselves in their discussion, a (challenging) perspective, and remains out of the scope of the paper.

In sum, in my opinion, removing "that fuels rapid adaptive changes" from the title, editing the abstract and the introduction accordingly, turning the results paragraph "TE mutations in adaptive genes" as supplementary anecdotal results, and modifying the discussion to state clearly the difference between solid results and speculative ideas that need to be further tested would shorten,

focus, and strengthen the paper, without comprominsing its attractiveness for readers.

Detailed comments:

* Line 150, I am not sure about the correct hyphenation pattern for the phrase "wild type derived interval", but I found the option "wild type-derived" particularly confusing.
* Supplementary methods, why fixing arbitrarily the deletion rate to zero for class I elements? It would make more sense to use the same model everywhere, with an expected estimate of deletion rates quite low for class I (such a result would give the reader a better confidence in this setting). Even if it probably works to detect combinations of parameters in agreement to the data, I found the statistical method (comparison of Kolmogorov-Smirnov goodness-of-fit statistics for each parameter combination) rather cumbersome and suboptimal. Given that the authors already have a simulation tool and that the number of parameters is limited to 2 or 3, using an ABC framework would probably require less work and would make it possible to provide support intervals for parameters values, which are missing from the authors' results. In the same paragraph, I think it would be useful to explain how the estimated excision rate could be larger than the transposition rate while generating a positive exponential growth.
* Sup fig 2f, typo in "Excission"

Signed: Arnaud Le Rouzic

Reviewer #3 (Remarks to the Author):

The authors describe interesting observations of the genomic distribution and impact of heritable mutations generated by TE mobilization. They demonstrate that transposition leads to an exponential increase of insertion mutations. In addition, these insertions seem to be preferential for specific genomic loci and chromatin states. Although these findings provide novel insight in the functioning of TEs and possibly generate new lines of investigation, there are a number of concerns that need to be addressed.

An obvious question is about the genetic resources they used for their investigations. I can understand that these lines and their accompanying data were available and were worth to study but perhaps a simpler crossing design involving the ddm1 mutant might have been more efficient. The authors need to provide at least a rationale of their followed approach and offset this to alternative strategies.

The manuscript is not always written in a comprehensible way and seems targeted to expert readers with many assumed knowledge. In addition, in parts the results are quite descriptive without realistic interpretation or explanation of findings. A good example of this is the paragraph from line 88-96 on page 4. Why would a TE map to one or multiple sequences and what determines the differences between the TE families?

In line with this on page 5. How can DNA transposons, such as VANDAL21 and ATENSPM3, that follow a cut and paste propagation lead to an exponential spread of TEs in subsequent generations? Similarly, how can ATENSPM3 transposons share mutations in multiple insertions per epiRIL and how can this number expand in later generations without copy multiplication? What causes the difference in excision rates between VANDAL21 and ATENSPM3?
I am clearly not an expert in TEs but if the authors state in their introduction that TEs fall into two classes: DNA transposons, that use a cut and paste mechanism, and retrotransposons that move via a RT-RNA intermediate I am confused how the first class can ever increase in number in any situation?

Related to this section of the manuscript is figure 2. Here the legend and the manuscript mention 10 epiRILs but the graphs in panel a only seem to show data of five lines. The legend itself is not very explanatory and only with the main text at hand the figure can be understood.

In line 164 it is concluded that excision of TEs has a larger effect than integration. How would the authors explain this observation, which mechanisms are in play?

The sections on TEs targeting to specific chromatin signatures and H2A.Z guided integration are in my view the most informative part of the manuscript. However, the observation of preferential insertion of TEs in adaptive trait genes is interesting but also quite arbitrary. It is hard to define genes as adaptive or not. Moreover, the examples given are cherry picking and might be misleading. Here, a less speculative attitude would have been preferred. The authors might tone down their message somewhat and present their results in a more neutral fashion. Showcasing their examples is fine and a nice demonstration of the consequences of their findings but it should be brought with care and nuance to avoid overstating their conclusions.

Finally, perhaps a word of caution in the discussion that these results were obtained in an artificial system with increased transposition rates. What would be the contribution of TEs to natural variation in wild type natural settings?

Dear Editor,

We thank the reviewers for their positive appraisal of our manuscript and the points they raised, which we have answered as detailed below. We believe that as a result, the revised manuscript is substantially improved and we are grateful to the reviewers for this.
We thank you for your time and your consideration and we look forward to hearing from you soon.

Best regards,

Leandro Quadrana and Vincent Colot

Reviewer #1 (Remarks to the Author):

This is well-executed study of how active transpsons behave en planta, and provides insight into how transposon excision/insertion can impact gene function and trait evolution. Using the EpiRIL system developed by the Colot lab, transposon properties are experimentally tested and modeled, including which transposons are active (three dominant transposon families), excision and transposition rates, insertion preference relative to gene and epigenetic features, and the natural limits on transposon accumulation by epigenetic silencing. One strength of this manuscript is that in addition to identifying correlative relationships, such as the preferential insertion of Ty1/copia at H2A.Z sites, they also experimentally confirm the observation, in this case by crossing the appropriate EpiRIL with and H2A.Z altered mutant (hta9 hta1 mutant). Additionally, the last section discussing the impact of ATCOPIA78/93 on gene expression and the example of the Ag-0 FLC/ATCOPIA78 helps to show that the synthetic EpiRIL system can provide insight into what happens in wild populations.

The only major criticisms I have is that they should provide more of an introduction to the EpiRIL system, in particular the fact that each EpiRIL has a mosaic genome composed of the ddm1 and wt intervals. It is mentioned when relevant to specific analysis, but would be helpful to have it laid out more explicitly as this is an important property of the EpiRILs that distinguishes them from a wild type plant. As part of this, I think it would be helpful to have some analysis of the rate of insertion of the three major transposons into the wild type and ddm1 intervals. The Fig S4f does describe this for ATCOPIA93, but I think it would be helpful to do so explicitly and earlier as it was a question I had while reading through the manuscript. I doubt that preferential "epi-type" insertion would confound any of the major conclusions of the manuscript, but I would like to these discussed more directly with some basic analysis.

We thank again this reviewer for his/her comments. To answer his/her major criticisms, we have expanded the description of the epiRIL population (lines 68-74) and have added a paragraph to present the insertion patterns observed in wildtype- and *ddm1*-derived chromosomal intervals (lines 158-165; Supplementary Fig. 4a). This new analysis confirms that euchromatin is the preferred substrate for the integration of *VANDAL21*, *ATENSPM3* and *ATCOPIA93*. Note also that in the original as well as the revised version of the manuscript, we indicated that “annotated TE sequences were more often hit by *ATCOPIA93* when inherited from the hypomethylated *ddm1* parent” (lines 207-212 in the revised manuscript).

Smaller issues:
Fig S5c - The description of S5c seems to be missing from the figure caption.
We apologize for this error, which is now corrected.

113 (Figure S2B). Different format than other places.

This has been corrected

208-211 "As a result, there were almost three times as many essential genes targeted by ATCOPIA93 in mutant than in wild type seedlings (Fig. 4b). These results indicate that by targeting H2A.Z-containing nucleosome arrays, ATCOPIA93 avoids essential genes and thus minimizes the risks of being lost together with its host."
The observed lower rate of targeting of essential genes in wild type is a really interesting observation, but I don't quite get the proposed mechanism as it relates to H2A.Z. Does the lower H2A.Z levels in the mutants result in more distributed insertion events thus increasing the essential gene targeting? Do essential genes have higher H2A.Z in the mutant and thus higher targeting? I think a sentence or two to describe exactly what the authors think is happening here would be helpful.

Indeed, our results indicate that *ATCOPIA93* insertions sites are more uniformly distributed across annotations and chromatin features in the absence of H2A.Z. We have clarified this point in the revised manuscript (lines 223 to 225).

137-47 - This is a really interesting section, but I'm wondering if they could more extensively test the original siRNA/Tn-load hypothesis described in Mari-Ordonez et al. That is, if they did a finer grain analysis of copy number and Tn silencing in EpiRIL458 between the F8-F16 (Fig S3b) it would help clarify if there is a specific copy number range that triggers silencing of all targets as described in Mari-Ordonez et al., or is silencing more gradually acquired across a few generations. This isn't critical to the current study, but would provide strong, independent confirmation of a very interesting and important model of transposon silencing.

Our modelling results are consistent with the experimental data reported by Mari-Ordonez et al 2013 (i.e. Fig. 3f and Fig. 4c-h), which establish that *ATCOPIA93* copies simultaneously undergo an ‘all-or-nothing’ methylation process, leading to a sharp onset of *ATCOPIA93* silencing in one generation. We have now obtained an independent confirmation of this point, by analyzing copy number, DNA methylation and RNA levels of *ATCOPIA93* in successive generations for epiRIL394, which we used for our phenotypic analyses (Supplementary Fig. 3c and lines 150-153 in revised manuscript).

Reviewer #2 (Remarks to the Author):

Review of "Transposition generates high rates of targeted mutations that fuel rapid adaptive changes" by L. Quadrana et al., NSCOMMS-19-13366.

This paper reports the results of a mutation accumulation experiment in A. thaliana, in which mutations were induced by an epigenetic induction of transposable element activity. The authors observed that (i) new TE insertions tend to accumulate in their experimental lines, (ii) this accumulation was exponential, supporting the idea that the newly generated insertions were themselves able to transpose, (iii) TE insertions were not uniformly distributed in the genome, but appeared to be more frequent around genes, (iv) different TE families display different insertion distribution patterns related to different chromatin signatures, (v) in particular, Ty1/copia retrotransposons were associated with a specific histone variant, H2A.Z, which is a marker of environmentally-induced genes.

Although each of these results is not totally unheard from the literature, the size of the experiment, the precision of the molecular analysis, and the accumulation of consistent evidence clearly justifies the publication of this study in a first-rank journal. As far as I know, this is the best TE-induced mutation accumulation study in any organism, and the authors' endeavor at providing a deep and detailed analysis of each result is remarkable (at the cost of overcrowded stamp-sized figures). As a non-specialist in epigenetics and chromatin states, I will focus my review on population genetics considerations, which should not be interpreted as a lack of interest for the main results of this study.

We thank Arnaud Le Rouzic for his enthusiastic comments.

On the down side, I found the "adaptation" part of the manuscript much less robust than all other claims, and I am not convinced that this deserves so much emphasis (one third of the title), given the weakness of empirical evidence. If I interpret correctly the results, the authors have shown that in one epiRIL selected for its richness in TE insertions in plastic genes, TEs tend to inhibit gene expression at generation F8, but not at generation F17. The authors further showed that this change in expression is associated with the expected phenotypic consequences for one gene. Independently, the authors noticed that a preexisting Copia insertion in the FLC gene was polymorphic in A. thaliana, and that this insertion was associated with a change in gene expression in specific environmental conditions (heat stress). The authors' hypothesis is that this insertion could be present in some populations due to local adaptation, which is difficult to assess without additional evidence.
From these two observations ((i) in at least one epiRIL, some TE insertions located in plastic genes can decrease temporarily gene expression, and (ii) there is a natural TE insertion that could be associated with local adaptation), I do not think it is fair to conclude that "targeted mutations fuel rapid adaptive changes". The idea that there might be an adaptive process associated with the chromatin state TE insertion preference is a hypothesis proposed by the authors as a possible interpretation for their results, and should not be presented as a result of the study, which is about targeted insertions. In my opinion, this hypothesis as such is rather vague, and seems to stem into an adaptationist program rather than in modern population genetics (adaptive "mutator" loci are highly unlikely in recombining organisms). In particular, some of the claims related to the adaptive potential of the reported mechanisms are vague and remain unchallenged throughout the manuscript. For instance, line 62, "endows TEs with a unique ability to accelerate the generation of adaptive mutations while minimizing the mutational load" does not seem to rely on any direct evidence. In the same

way, the discussion paragraph lines 288 - 294 states that "retrotransposons have been turned into epigenetically and environmentally sensitive engines of adaptive innovation", and that "such an evolutionary scenario provides a plausible explanation for the invasive success of this superfamily". A necessary condition for natural selection to favor Copia invasion is that the effects of TE insertions are in average more beneficial than deleterious, which I do not really find "plausible". In any case, testing adaptation as a consequence and/or a cause of TE specific insertion patterns is, as stated by the authors themselves in their discussion, a (challenging) perspective, and remains out of the scope of the paper.

In sum, in my opinion, removing "that fuels rapid adaptive changes" from the title, editing the abstract and the introduction accordingly, turning the results paragraph "TE mutations in adaptive genes" as supplementary anecdotal results, and modifying the discussion to state clearly the difference between solid results and speculative ideas that need to be further tested would shorten, focus, and strengthen the paper, without comprominsing its attractiveness for readers.

We also really appreciate these criticisms, which are fair. As a consequence, we have changed the title, abstract, introduction and discussion to remove any unsupported claim. We have also been very careful in distinguishing facts from speculations. As a result, we believe that the revised manuscript provides a more balanced view without compromising the far-reaching implications of our findings, thus preserving its attractiveness.

Detailed comments:

** Line 150, I am not sure about the correct hyphenation pattern for the phrase "wild type derived interval", but I found the option "wild type-derived" particularly confusing.*

We revised the manuscript so as to avoid this confusing hyphenation altogether.

** Supplementary methods, why fixing arbitrarily the deletion rate to zero for class I elements? It would make more sense to use the same model everywhere, with an expected estimate of deletion rates quite low for class I (such a result would give the reader a better confidence in this setting). Even if it probably works to detect combinations of parameters in agreement to the data, I found the statistical method (comparison of Kolmogorov-Smirnov goodness-of-fit statistics for each parameter combination) rather cumbersome and suboptimal. Given that the authors already have a simulation tool and that the number of parameters is limited to 2 or 3, using an ABC framework would probably require less work and would make it possible to provide support intervals for parameters values, which are missing from the authors' results. In the same paragraph, I think it would be useful to explain how the estimated excision rate could be larger than the transposition rate while generating a positive exponential growth.*

Although we agree that extra modeling could provide additional information, we feel that this is beyond the scope of the present manuscript. However, we have re-run the simulations for *ATCOPIA93* by including excision of transposed copies as an extra parameter. This enabled us to confirm that our model constructed with a transposition rate of ~0.6 transposition events/copy/generation and no excision remains within the goodness-of-fit peak (See figure below). However, other well-fitting models were obtained, which imposed high transposition and excision rates. As expected, these rates scale linearly with a 2:1 relationship leading to an effective replicative rate of ~0.6, as estimated in our simplest model. Thus, we believe that

incorporating the unrealistic excision parameter to the model for *ATCOPIA93* will add confusion rather than confidence, without contributing any novel insights.

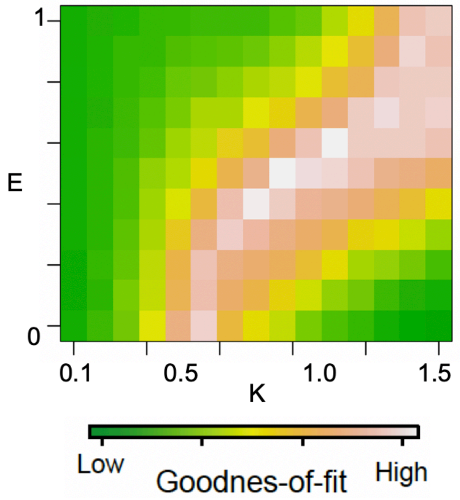

Goodness-of-fit between observed and simulated data obtained under a "transposon" model and with different transposition (K) and excission (E) rate values.

The statistical method we have used may not be optimal, but again, we don't see the point of refining it further or using an alternative one in the context of our current work. Indeed, this method (multivariate Kolmogorov-Smirnov test, also known as "Peacock test") was designed explicitly to assess the goodness-of-fit of mathematical models to observed data (Peacock 1983. Mon. Not. R. Astron. Soc.; Fasano and Franceschini 1987, *Monthly Notices of the Royal Astronomical Society*) and has been implemented successfully across diverse scientific disciplines (e.g. Bruns et al 2014. *Nature Medicine*; Barrick et al 2010, *Mol. Biol. Evol*; Michele et al, 2007. *Coastal Engineering*; Spergel et al., 1987 *Science*).

As to the last concern, we would like to emphasize that we defined the excision rate as the probability to lose the donor copy during non-replicative mobilization. Thus, an excision rate of 0.92 per transposition event for *ATENSPM3* implies that 8% of mobilization events appears to be replicative, which will lead, by definition, to a cumulative increase in the number of active copies. We have clarified this point in the revised manuscript (lines 134-137 of the main text and lines 455-456). One mechanistic explanation for these observations is that although mobilization of some DNA transposons, such as *VANDAL* or *ATENSPM3*, involves excision, double-stranded gap repair can be used to replace the excised copy with that present on the homolog or the sister chromatid, in case of post-replicative mobilization (Engels et al 1990, *Cell*). We have also discussed this point in the revised manuscript (lines 132-134 of the revised manuscript).

Excissions events repaired by
* Sup fig 2f, typo in "Excission"

This has been fixed.

Signed: Arnaud Le Rouzic

Reviewer #3 (Remarks to the Author):

The authors describe interesting observations of the genomic distribution and impact of heritable mutations generated by TE mobilization. They demonstrate that transposition leads to an exponential increase of insertion mutations. In addition, these insertions seem to be preferential for specific genomic loci and chromatin states. Although these findings provide novel insight in the functioning of TEs and possibly generate new lines of investigation, there are a number of concerns that need to be addressed.

We thank this reviewer for his/her positive comments.

An obvious question is about the genetic resources they used for their investigations. I can understand that these lines and their accompanying data were available and were worth to study but perhaps a simpler crossing design involving the ddm1 mutant might have been more efficient. The authors need to provide at least a rationale of their followed approach and offset this to alternative strategies.

By design, the epiRILs are mutation accumulation lines, which are the gold standard for the study of the mutational process (Lynch et al 2016, Nat. Rev. Genet.). As we now state explicitly in the revised manuscript, "the epiRIL population constitutes an ideal system to study TE mobilization in an essentially wild-type context and to compare insertion patterns in chromosomal intervals derived from the *ddm1* and wild-type parents" (lines 72-74).

The manuscript is not always written in a comprehensible way and seems targeted to expert readers with many assumed knowledge. In addition, in parts the results are quite descriptive without realistic interpretation or explanation of findings. A good example of this is the paragraph from line 88-96 on page 4. Why would a TE map to one or multiple sequences and what determines the differences between the TE families?

Typically, TE families contain multiple members, a small fraction of which are full-length. Among the latter, only one or a few copies are transpositionally competent. Low levels of sequence polymorphisms usually enable one to recognize these active copies from their inactive counterparts.
We have revised the manuscript to the best of our ability to explain all observations and analyses for a broad readership. In addition to providing an extended description of the epiRIL system, we have added several clarifications throughout the manuscript.

In line with this on page 5. How can DNA transposons, such as VANDAL21 and ATENSPM3, that follow a cut and paste propagation lead to an exponential spread of TEs in subsequent generations? Similarly, how can ATENSPM3 transposons share mutations in multiple insertions per epiRIL and how can this number expand in later generations without copy multiplication? What causes the difference in excision rates between VANDAL21 and ATENSPM3?
I am clearly not an expert in TEs but if the authors state in their introduction that TEs fall into two classes: DNA transposons, that use a cut and paste mechanism, and retrotransposons that move via a RT-RNA intermediate I am confused how the first class can ever increase in number in any situation?

We defined the excision rate as the probability to lose the donor copy during non-replicative mobilization. Thus, an excision rate of 0.92 per transposition event for *ATENSPM3* implies

that 8% of mobilization events appears to be replicative, which will lead, by definition, to a cumulative increase in the number of active copies. We have clarified this point in the revised manuscript (lines 134-137 of the main text and lines 455-456). One mechanistic explanation for these observations is that although mobilization of some DNA transposons, such as *VANDAL* or *ATENSPM3*, involves excision, double-stranded gap repair can be used to replace the excised copy with that present on the homolog or the sister chromatid, in case of post-replicative mobilization (Engels et al 1990, *Cell*). We have also discussed this point in the revised manuscript (lines 132-134 of the revised manuscript).

Related to this section of the manuscript is figure 2. Here the legend and the manuscript mention 10 epiRILs but the graphs in panel a only seem to show data of five lines. The legend itself is not very explanatory and only with the main text at hand the figure can be understood.

We have now clarified the legend of Fig. 2a.

In line 164 it is concluded that excision of TEs has a larger effect than integration. How would the authors explain this observation, which mechanisms are in play?

A mechanistic understanding of this observation would require considerable work, and we feel that possible explanations would be too speculative at this stage.

The sections on TEs targeting to specific chromatin signatures and H2A.Z guided integration are in my view the most informative part of the manuscript. However, the observation of preferential insertion of TEs in adaptive trait genes is interesting but also quite arbitrary. It is hard to define genes as adaptive or not. Moreover, the examples given are cherry picking and might be misleading. Here, a less speculative attitude would have been preferred. The authors might tone down their message somewhat and present their results in a more neutral fashion. Showcasing their examples is fine and a nice demonstration of the consequences of their findings but it should be brought with care and nuance to avoid overstating their conclusions.

We have changed the text to avoid defining genes as adaptive or not, although environmentally responsive genes have been considered as strong candidates for adaptation (Hoffman and Willi 2011, *Nature Rev. Genet*; Hancock et al 2010, Science; Grivet et al 2010, *Mol. Biol. Evol.*). Furthermore, we have changed the title, abstract, introduction and discussion to remove any unsupported claim.

Finally, perhaps a word of caution in the discussion that these results were obtained in an artificial system with increased transposition rates. What would be the contribution of TEs to natural variation in wild type natural settings?

We further stress in the discussion the importance of this point (lines 314 to 316 of the revised manuscript).

REVIEWERS' COMMENTS:

Reviewer #1 (Remarks to the Author):

The authors have addressed all of my criticisms, both major and minor, with additional text and new analysis. I have no additional concerns.

Reviewer #2 (Remarks to the Author):

Review of "Transposition favors the generation of large effect mutations that may facilitate rapid adaptation" by L. Quadrana and colleagues, NSCOMM-19-13366A.

This is a revision of a previous manuscript that was largely acknowledged by the reviewers as an important contribution to the field. I will not list again the reasons why this study deserves to be published in a wide-scope journal. Yet, several points were raised by the reviewers, including (i) the need to expand the description of the principles of EpiRIL experiments, (ii) the fact that the limit between results and speculations was not clearly defined, and (iii) the ms implicitly assumed a deep understanding of transposable element biology, which could be a problem for naive readers.

The manuscript has been revised in order to minimize these issues, without affecting the structure or the results. Since these remarks were related to the style and readability of the paper, and not to the scientific results, I think it is reasonable to consider them as fixed and not delay the publication further.

I acknowledge the authors' detailed answer on a minor point I made about model fitting and parameter estimates. I still think that a statistical framework providing not only a point estimate, but also a confidence/support interval around the estimate would have been more appropriate (such an interval would have probably included zero for the deletion rate of the ATCOPIA93 case for instance, which would have fixed the disturbing non-zero point estimate of the excision rate for a TE that is not supposed to excise). Nevertheless, this dispute is on a minor and dispensable detail of the ms, and I agree with the authors that fixing it would not make a big difference. I assume that the natural conclusion would be that support intervals are quite large, reflecting the difficultu to estimate both duplication and excision rates from a TE amplification experiment (which is by itself not surprising).

Signed: A. Le Rouzic

Reviewer #3 (Remarks to the Author):

I feel that the points raised in the previous round of review have been satisfactorily addressed and I have no further comments.

Reviewer #1 (Remarks to the Author):
The authors have addressed all of my criticisms, both major and minor, with additional text and new analysis. I have no additional concerns.

We are glad to see that we addressed all criticisms of reviewer 1.

Reviewer #2 (Remarks to the Author):
Review of "Transposition favors the generation of large effect mutations that may facilitate rapid adaptation" by L. Quadrana and colleagues, NSCOMM-19-13366A.
This is a revision of a previous manuscript that was largely acknowledged by the reviewers as an important contribution to the field. I will not list again the reasons why this study deserves to be published in a wide-scope journal. Yet, several points were raised by the reviewers, including (i) the need to expand the description of the principles of EpiRIL experiments, (ii) the fact that the limit between results and speculations was not clearly defined, and (iii) the ms implicitly assumed a deep understanding of transposable element biology, which could be a problem for naive readers.

The manuscript has been revised in order to minimize these issues, without affecting the structure or the results. Since these remarks were related to the style and readability of the paper, and not to the scientific results, I think it is reasonable to consider them as fixed and not delay the publication further.

We thank again reviewer 2 for his positive comments.

I acknowledge the authors' detailed answer on a minor point I made about model fitting and parameter estimates. I still think that a statistical framework providing not only a point estimate, but also a confidence/support interval around the estimate would have been more appropriate (such an interval would have probably included zero for the deletion rate of the ATCOPIA93 case for instance, which would have fixed the disturbing non-zero point estimate of the excision rate for a TE that is not supposed to excise). Nevertheless, this dispute is on a minor and dispensable detail of the ms, and I agree with the authors that fixing it would not make a big difference. I assume that the natural conclusion would be that support intervals are quite large, reflecting the difficultu to estimate both duplication and excision rates from a TE amplification experiment (which is by itself not surprising).

As we stated in our first response to reviewer 2, additional modeling is beyond the scope of the present study. We are pleased to see that reviewer 2 states that this dispute is on a minor and dispensable detail of the manuscript and that he agrees with us that fixing it would not make a big difference.

Signed: A. Le Rouzic

Reviewer #3 (Remarks to the Author):
I feel that the points raised in the previous round of review have been satisfactorily addressed and I have no further comments.

We are glad to see that we satisfactorily addressed all criticisms raised in the previous version of the manuscript.
